# Sustainable nanofiltration of industrial wastewater using waste-derived-graphene oxide-modified polyamide membranes: machine learning toward a smart portable water treatment unit

**DOI:** 10.1039/d6ra05062j

**Published:** 2026-09-03

**Authors:** Montassar T. Bouzidi, Mariem M. BenSlimene, Tawfik A. Saleh

**Affiliations:** a Department of Chemistry, King Fahd University of Petroleum & Minerals Dhahran 31261 Saudi Arabia tawfikas@hotmail.com tawfik@kfupm.edu.sa https://faculty.kfupm.edu.sa/CHEM/tawfik/research.html; b Interdisciplinary Research Center for Advanced Materials, King Fahd University of Petroleum & Minerals Dhahran 31261 Saudi Arabia

## Abstract

Industrial wastewater treatment poses significant challenges because effluents vary in composition, containing dissolved salts, organic contaminants, and heavy metals. Nanofiltration (NF), employing thin-film composite (TFC) membranes, offers an energy-efficient separation platform capable of rejecting multivalent ions, organics, and dissolved metals. Here, graphene oxide (GO) was synthesized from waste graphite *via* a modified Hummers' method, functionalized with imidazole groups, and incorporated into polyamide TFC membranes to enhance rejection performance. The membranes were characterized, then evaluated for their efficiency in separating a mixture of pollutants in wastewater. To overcome limitations of one-factor-at-a-time experimentation, filtration experiments spanning ranges of pressure and feed composition were used to build a hybrid machine learning (ML) framework. Target-specific regression models, including *k*-nearest neighbors (*k*NN), Extreme Gradient Boosting (XGBoost), and support vector regression (SVR), and random forest (RF), were evaluated and compared based on their predictive accuracy and generalization performance. The optimized models achieved good predictive accuracy: *R*^2^ = 0.879 (*k*NN) for metal-ion permeate concentration, *R*^2^ = 0.998 (XGBoost) for organic-pollutant permeate concentration, and *R*^2^ = 0.983 (SVR) for salt permeate concentration. Model-guided analysis identified operating regimes that maintained high rejection while preserving acceptable permeate flux. The validated framework was integrated into a portable bench-top NF unit equipped with a web-based predictive interface, enabling real-time guidance under variable feed conditions. This hybrid ML/experimental approach provides a scalable, data-informed pathway for industrial wastewater recycling, advancing dual sustainability through graphite waste valorization and efficient water reuse.

## Abstract 2

Decentralized wastewater treatment uses GO-modified NF membranes with target-specific ML models. This integrated ML-NF framework provides predictive process optimization, cutting costs and improving water sustainability at a demonstration scale.

## Graphical abstract

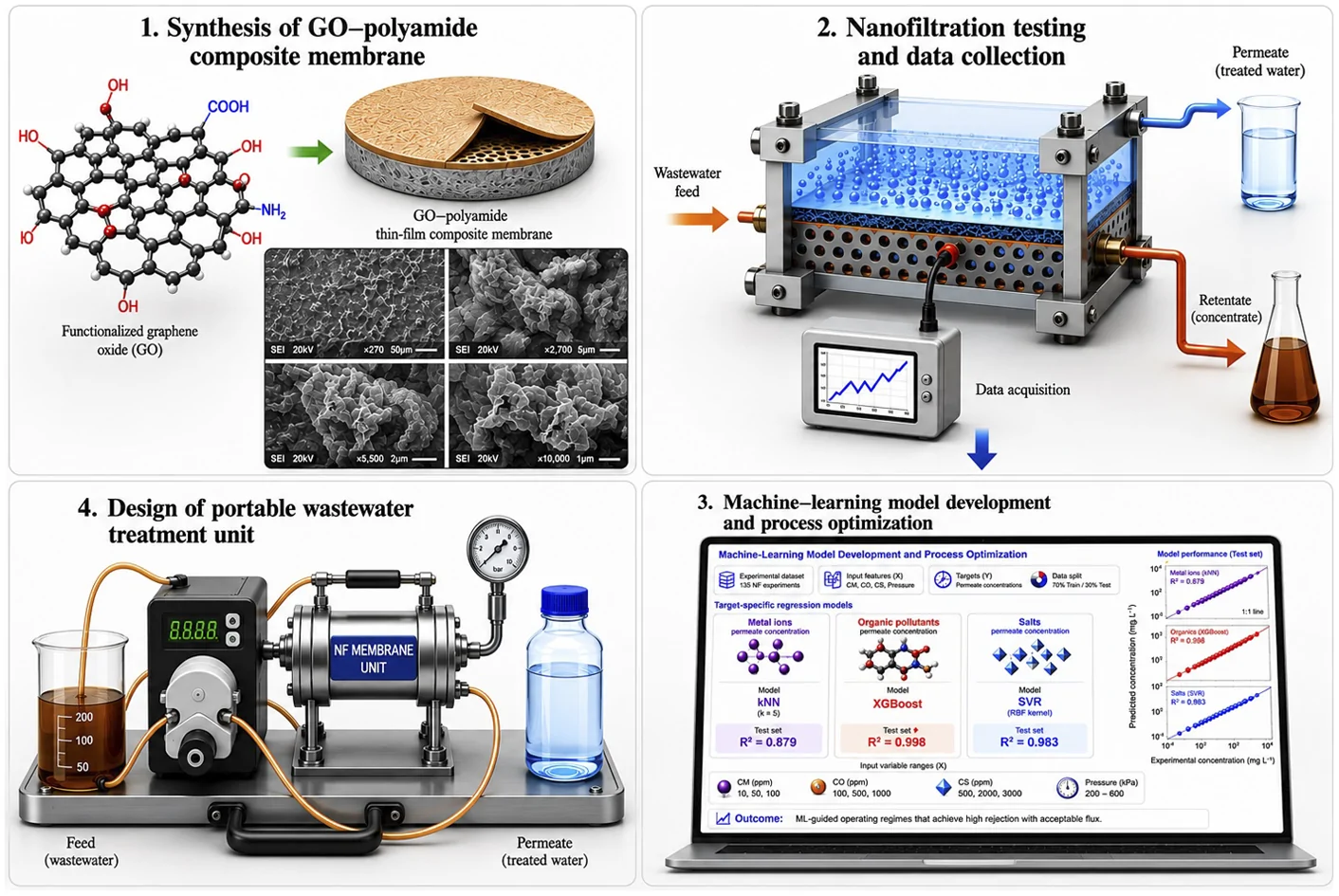

## 1 Introduction

Industrial wastewater recycling is challenging because effluents often contain complex mixtures of dissolved salts, organic contaminants, and heavy metals at fluctuating concentrations. Adaptable separation methods are essential to meet stringent discharge regulations. Nanofiltration (NF) is mostly operated at lower pressures than reverse osmosis. Conventional NF systems generally operate at around 3–20 bar. However, highly permeable low-pressure NF membranes can operate at pressures as low as 2 bar while retaining multivalent ions. Therefore, the present study investigated a pressure range of 200–600 kPa (2–6 bar) to evaluate the performance of the developed GO-modified polyamide membrane under low-pressure NF conditions. However, the performance of NF membranes is captured by highly interdependent variables, including feed composition, transmembrane pressure, membrane surface chemistry, and hydrodynamic conditions, which generate nonlinear relationships between permeate flux, contaminant rejection, and fouling behavior. Parameter optimization through conventional one-factor-at-a-time experimentation is therefore impractical for identifying true global operating optima, a limitation widely recognized as a bottleneck in advanced water treatment design.^1^

To address this operational complexity, it is necessary to develop efficient materials, design high-performance membranes, and advance process optimization. Thin-film composite (TFC) membranes, prepared by interfacial polymerization of monomers to form a polyamide layer on a porous support, are considered a robust platform that combines permeability, mechanical stability, and selectivity.^2–4^ Building on this foundation, the incorporation of functional nanomaterials into polyamide systems has been explored to improve membrane performance. Graphene oxide (GO) is a promising material for improving surface hydrophilicity, enhancing fouling resistance, and increasing rejection efficiency without severely compromising flux.^5,6^ GO-modified membranes can outperform conventional materials under controlled conditions.^7–9^ However, challenges remain regarding the application of these advanced membranes under realistic and variable water conditions.^10^ The distinction between material capability and process optimization is highlighted by the fact that even high-performance membranes may perform poorly if operated outside their optimal range.^11^

The gap between operational efficiency and membrane performance potential has motivated growing interest in data-driven modeling approaches. Machine learning (ML) has shown considerable promise in water applications where system behavior is captured by multivariate, nonlinear interactions that are difficult to capture using first-principles models.^12,13^ In membrane science, ML has been widely used for performance prediction, fouling analysis, and system monitoring, often achieving high apparent accuracy.^14–17^ However, several limitations restrict the practical utility of existing ML models, which are often trained and validated on idealized datasets, raising concerns about robustness under unseen operating conditions.^18–20^ In many cases, ML is treated as a black-box predictor, offering limited interpretability and little actionable guidance for process engineers seeking to optimize operating conditions.^21–23^ These limitations have hindered the translation of ML from research to reliable decision-support tools in industrial membrane processes. The development of a sustainable wastewater treatment system requires the integration of resource-efficient nanomaterials, energy-efficient processes, and data-driven optimization.

Here, GO derived from waste graphite provides a circular pathway for valorizing carbon-rich residues into functional membranes.^24,25^ Coupled with the moderate energy demand of NF and the use of ML to reduce experimental redundancy, this combination offers a sustainable and scalable strategy for industrial wastewater treatment. This work aims to bridge the gap between material innovation and process optimization by coupling the sustainable synthesis of GO-modified polyamide TFC membranes (GO-PA TFC) with a validated, target-specific ML framework for NF process optimization. Specifically, we synthesize and characterize GO-PA TFC membranes and then develop predictive ML models for three key performance indicators: permeate concentrations of metal ions (Y1), organic pollutants (Y2), and salts (Y3). Four machine-learning algorithms, *k*-nearest neighbors (*k*NN), support vector regression (SVR), Random Forest (RF), and Extreme Gradient Boosting (XGBoost), were evaluated to investigate instance-based, kernel-based, and ensemble learning paradigms, enabling a comprehensive assessment of the most suitable learning strategy for predicting each target variable according to its underlying transport behavior. The best performing model for each target was subsequently employed to predict membrane performance and identify interpretable optimal operating conditions that maximize permeate productivity while maintaining high solute rejection. This study thus integrates advanced membrane materials with data-driven process optimization toward scalable, intelligent NF-based water treatment.

## 2 Experimental methodology

### 2.1 Materials

Polyvinylidene fluoride (PVDF, (C_2_H_2_F_2_)_*n*_) pellets were purchased from Shanghai Aladdin Reagent Co. and used to prepare the membrane support. *N*,*N*-Dimethylformamide (DMF, HCON(CH_3_)_2_) served as the casting solvent, and polyvinylpyrrolidone (PVP) was added as a pore-forming agent. 1,3,5-Benzenetricarbonyl trichloride (trimesoyl chloride, TMC, 98%, C_9_H_3_Cl_3_O_3_), *m*-phenylenediamine (MPD, 99%, C_6_H_8_N_2_), and hexane (C_6_H_14_), which were used for selective-layer fabrication *via* interfacial polymerization, were obtained from Sigma-Aldrich. Sulfuric acid (H_2_SO_4_), nitric acid (HNO_3_), potassium permanganate (KMnO_4_), hydrogen peroxide (H_2_O_2_), 4-(1*H*-imidazole-4-yl)butan-1-amine (C_7_H_13_N_3_), and thionyl chloride (SOCl_2_) were purchased from Sigma-Aldrich and used for the synthesis of modified graphene oxide. Chemicals used for membrane performance evaluation were procured from Fisher Scientific and Sigma-Aldrich and included hydrocarbons such as isooctane ((CH_3_)_2_CHCH_2_C(CH_3_)_3_) and toluene (C_6_H_5_CH_3_); inorganic salts, including sodium chloride (NaCl), calcium chloride (CaCl_2_), and sodium sulfate (Na_2_SO_4_); and metal-ion sources, including lead nitrate (Pb(NO_3_)_2_) and cadmium nitrate (Cd(NO_3_)_2_). DI water was used throughout membrane preparation and filtration experiments.

### 2.2 Synthesis

#### 2.2.1 Graphene preparation

**Fig. 1:**
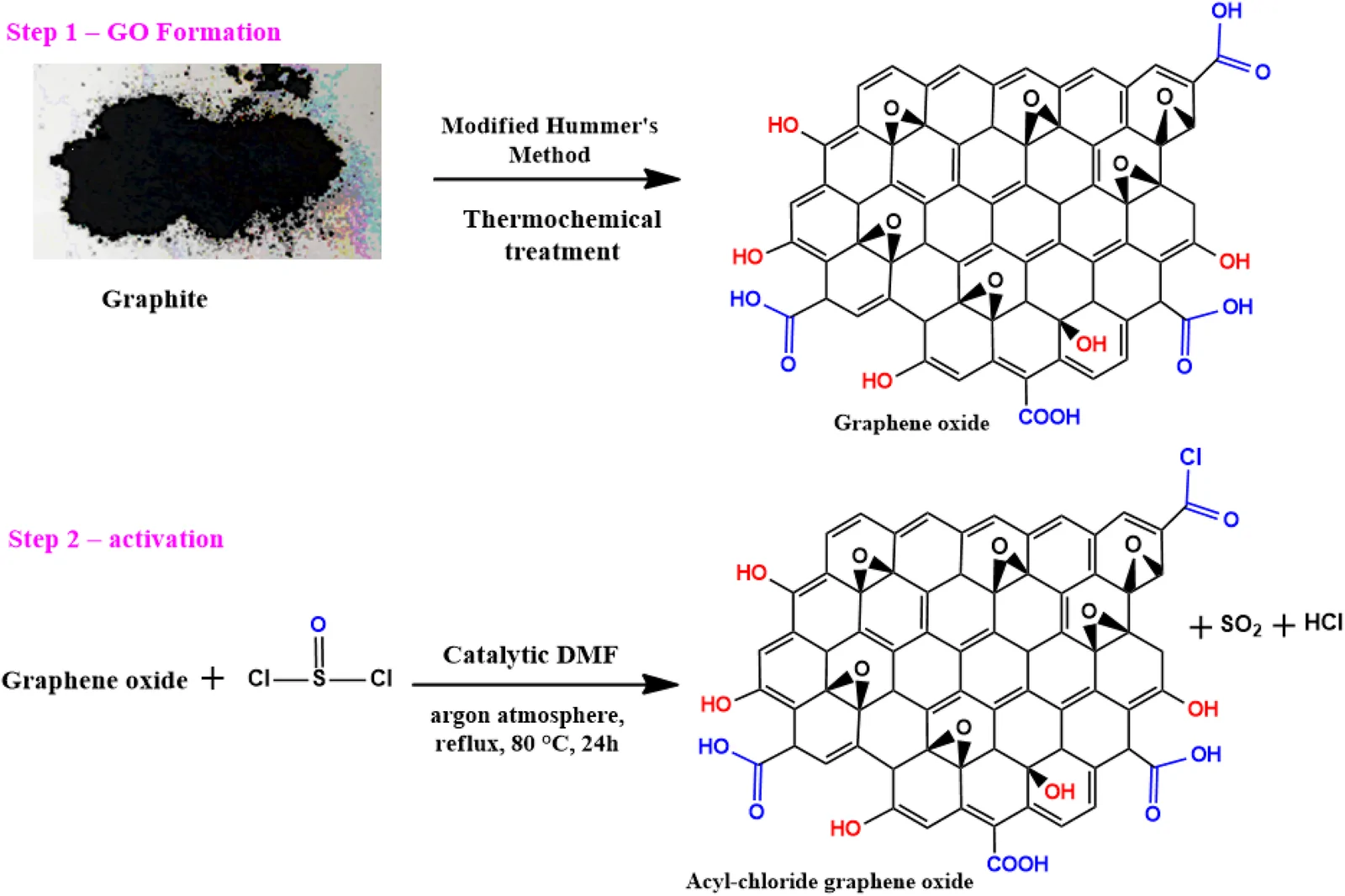
Scheme of (Step 1) graphene oxide (GO) synthesis, and (Step 2) acyl chloride activation using SOCl_2_.

Graphene oxide (GO) was prepared from graphite powder *via* a modified Hummers' method.^26^ This was followed by activation using nitric acid. In this step, 1 g of graphene oxide was treated with 80 mL of concentrated nitric acid at 90 °C for 3 h.^27^ After cooling, the mixture was separated by centrifugation at 10 000 rpm, as shown in Step 1, Fig. 1. The obtained graphene oxide (GO) was allowed to dry under vacuum at 60 °C to remove residual water and traces of nitric acid remaining on the surface. For modified graphene synthesis, the graphene oxide (GO, 500 mg) was placed in a dry round-bottom flask, and 20 mL thionyl chloride (SOCl_2_) containing 0.5 mL anhydrous DMF was added. The mixture was kept under stirring in an argon atmosphere and refluxed at 80 °C for one day, to convert the edge carboxylic acid (–COOH) groups of graphene oxide (GO) into highly reactive acyl chloride (–COCl) groups, facilitating subsequent functionalization.^28^ After completion, excess SOCl_2_ was removed under reduced pressure, and the resulting acyl chloride-functionalized GO (GO–COCl) was washed with dry dichloromethane to remove residual SOCl_2_, then dried under vacuum. The obtained GO–COCl was then dispersed in 30 mL of anhydrous DMF by sonication for 30 min, Step 2, Fig. 1.

#### 2.2.2 Synthesis of imidazole-modified graphene

**Fig. 2:**
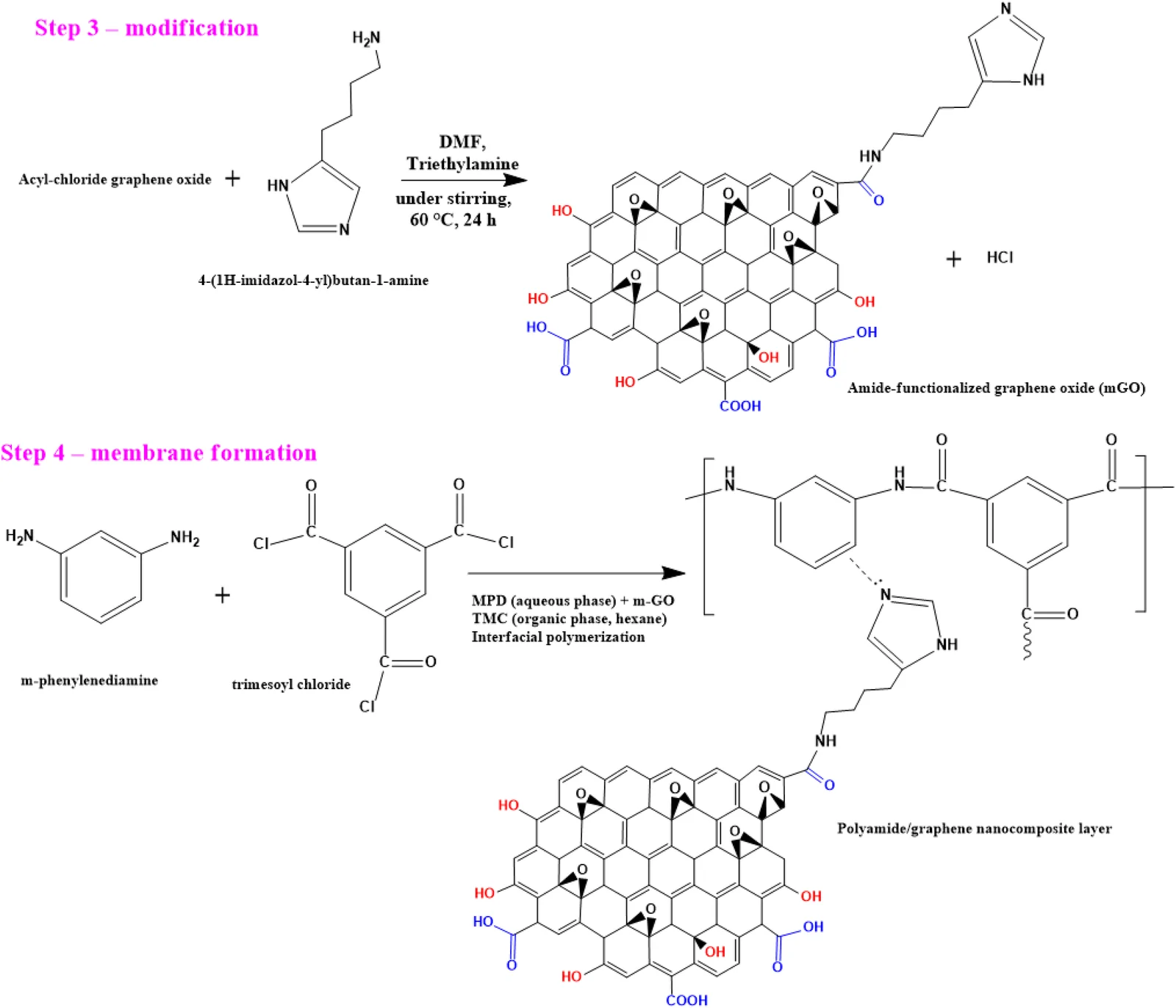
Illustration of the proposed incorporation of imidazole-functionalized GO into the polyamide selective layer (Step 3) possible amidation with 4-(1*H*-imidazole-4-yl)butan-1-amine to produce imidazole-modified graphene (mGO), and (Step 4) formation of graphene-modified polyamide membrane *via* interfacial polymerization.

Subsequently, 4-(1*H*-imidazole-4-yl)butan-1-amine (0.5 g) and triethylamine (1 mL, added to neutralize HCl produced during amidation) were added under argon. The system was stirred at 60 °C for 24 h to allow amidation between the acyl chloride groups on GO and the terminal primary amine (–NH_2_) of 4-(1*H*-imidazole-4-yl)butan-1-amine, Step 3, Fig. 2. The reaction preferentially occurs *via* the primary amine group (–NH_2_), as the imidazole nitrogen is less nucleophilic due to aromatic stabilization. After the reaction, the solid product was recovered by centrifugation and washed with methanol and deionized water to remove unreacted amine, triethylamine hydrochloride, and physically adsorbed species. The product was dried in a vacuum oven at 40–50 °C overnight to obtain amide-linked imidazole-functionalized graphene oxide.^29^

#### 2.2.3 Membrane support preparation

The support was prepared using the casting method by dissolving 2.4 g of polyvinylidene fluoride (PVDF) powder in 30 mL of DMF under magnetic stirring for 2 h. After complete dissolution, 0.4 g of polyvinylpyrrolidone was added as a pore-forming and structure-directing agent, and the solution was stirred at 400 rpm for 24 h. The resulting homogeneous solution was cast onto a glass plate with a 200 µm knife gap and then immersed in 500 mL of DI water to induce phase inversion. After 24 h, the membrane was transferred to fresh DI water for an additional 24 h to leach out residual solvent and additives, then dried under vacuum.^30^

#### 2.2.4 Preparation of graphene-modified membranes

The graphene-modified thin-film composite (TFC) membrane was fabricated by interfacial polymerization on the PVDF support. Prior to polymerization, the PVDF support was pretreated in 0.1 M HNO_3_ for 2 h to leach trace impurities and activate the pores, and was then rinsed with DI water until the eluate reached neutral pH. The membrane support was taped flat to a glass plate, and the surface was coated with an aqueous solution of 2 wt% *m*-phenylenediamine containing 200 mg of modified graphene. The membrane was then immersed in 0.6 wt% trimesoyl chloride in *n*-hexane for 15 s to initiate interfacial polymerization. This sequence was repeated ten times to build a defect-free selective layer. Excess solution was removed with a rubber roller, and the prepared membrane was cured at 55 °C for 30 min, as shown in Step 4, Fig. 2.

### 2.3 Testing setup and data acquisition

**Fig. 3:**
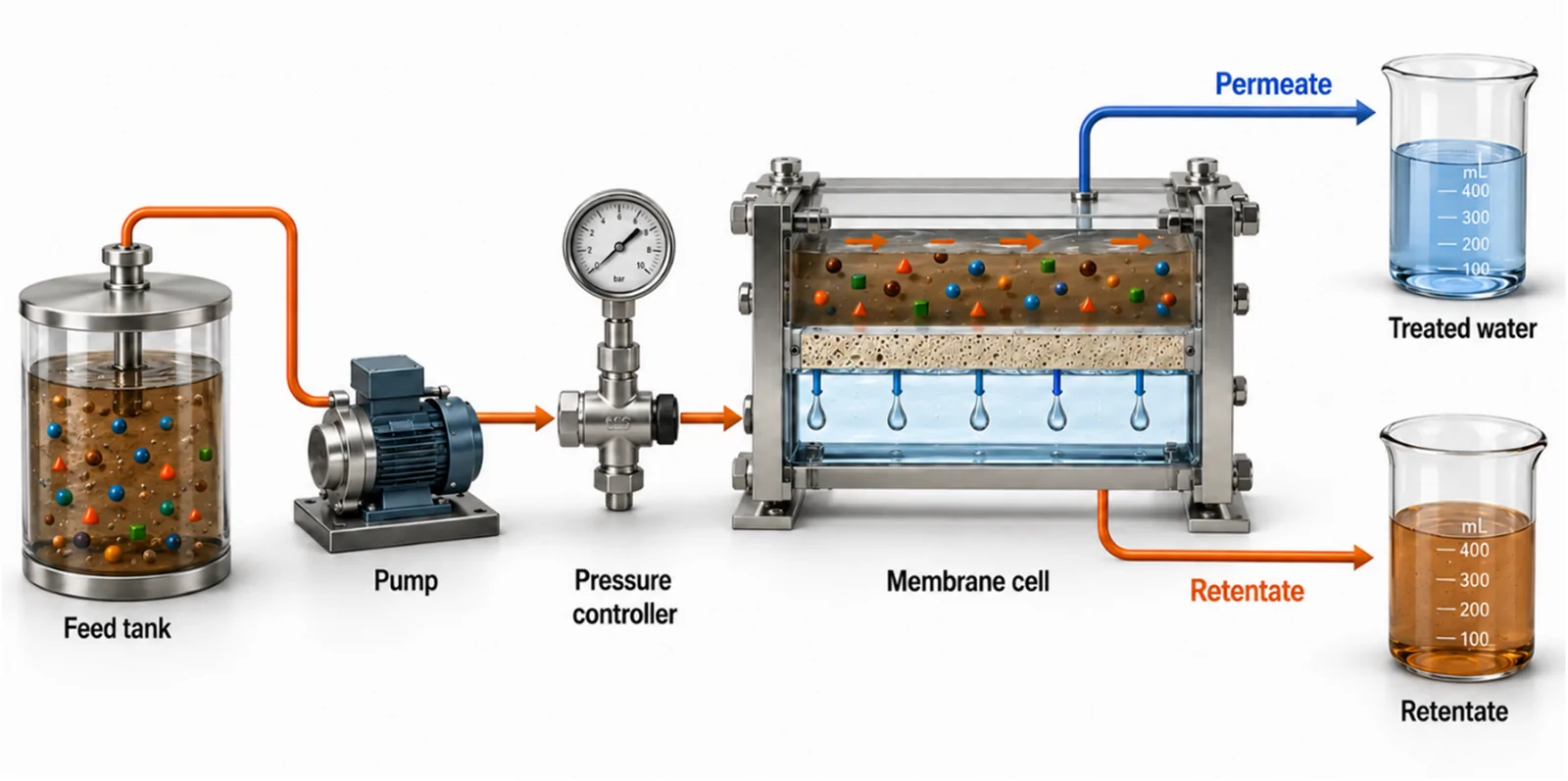
The design of the cross-flow nanofiltration setup used for membrane performance testing and operation.

A laboratory-scale crossflow nanofiltration (NF) system was assembled, consisting of a stainless-steel feed tank, a variable-speed pump, inline pressure gauges, a flat-sheet membrane module, and a permeate collection vessel. The membrane module provided an effective filtration area of 36 cm^2^. Transmembrane pressure was controlled by pump speed and monitored using the pressure gauge, whereas excess retentate was recirculated to the feed tank. The system was operated under steady cross-flow conditions, and the feed temperature was kept constant using an external temperature-control loop, Fig. 3. After reaching steady-state operation, feed and permeate samples were collected. A total of 135 filtration experiments were designed using a full-factorial combination of four input variables. The feed metal-ion concentration (CM) was varied at 10, 50, and 100 ppm, the organic contaminant concentration (CO) at 100, 500, and 1000 ppm, the salt concentration (CS) at 500, 2000, and 3000 ppm, and the operating pressure (*P*) at 200, 300, 400, 500, and 600 kPa. Combining the three concentration levels with the five pressure levels generated 3 × 3 × 3 × 5 = 135 unique operating conditions, providing a comprehensive dataset for machine-learning model development and validation. Metal-ion concentrations were analyzed by inductively coupled plasma optical emission spectrometry (ICP-OES), salt concentrations by ion chromatography (IC), and organic contaminant concentrations by gas chromatography-mass spectrometry (GC-MS). In the ML framework, experimentally controlled operating parameters were used as input variables and membrane performance metrics as output variables, Table S1.

## 3 ML methodology

### 3.1 Workflow overview

**Fig. 4:**
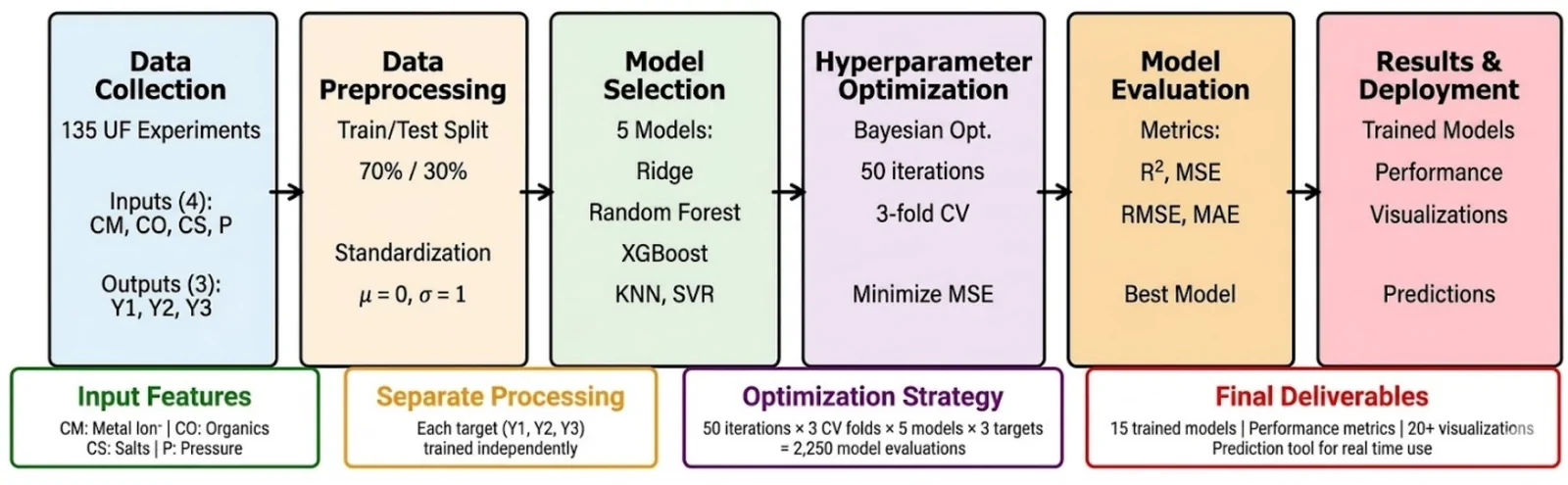
Workflow of the ML process for the prediction of NF membrane performance.

A structured ML framework was developed to model NF membrane performance from experimental data collected across a systematic range of operating parameters. The overall workflow, from data preparation to model evaluation, is illustrated in Fig. 4. The 135 experimental observations were split into 70% training and 30% testing subsets using a fixed random state to ensure reproducibility.

Before model training, all input variables were standardized to zero mean and unit variance. All data preprocessing steps were applied in a manner that prevents data leakage, with scaling parameters derived exclusively from the training subset and then applied to the test data. Separate regression models were developed for each target variable (Y1, Y2, and Y3) to account for differences in transport and separation mechanisms. This approach was used to prevent cross-target interference and enhance robustness in membrane-related modeling.^31,32^

### 3.2 Models evaluated

Four regression algorithms were selected to provide complementary learning paradigms capable of capturing the diverse transport mechanisms governing NF performance across three target variables (Y1–Y3). (i) *k*-Nearest neighbors (*k*NN): an instance-based (lazy) learner that estimates the output by averaging the responses of the *k* most similar training samples. *k*NN is non-parametric with respect to functional form and can naturally capture highly localized, nonlinear response surfaces that arise when rejection behavior changes abruptly with operating conditions. (ii) Support vector regression (SVR): a kernel-based method that maps inputs into a high-dimensional feature space *via* a radial basis function (RBF) kernel, enabling the capture of smooth, globally continuous response behaviors without requiring explicit feature engineering. SVR is effective when the target surface is regular but nonlinear, and is robust to outliers through its *ε*-insensitive loss formulation. (iii) Random Forest (RF): a bagged ensemble of decision trees trained on bootstrap samples with random feature subsets. RF reduces variance relative to individual trees, handles feature interactions implicitly, and provides a natural measure of feature importance. (iv) Extreme Gradient Boosting (XGBoost): a sequential boosting ensemble that corrects residuals of previous trees using gradient descent. XGBoost is particularly effective for structured tabular data with complex interactions and has demonstrated state-of-the-art performance in water treatment prediction tasks. This work pairs an expanded, independent experimental dataset with ML models using new GO physicochemical descriptors and prospective validation, providing predictive design insight compared to other studies.^33–36^ Ridge regression was additionally evaluated as a regularized linear baseline to determine whether nonlinear models provided improved predictive performance. Full mathematical formulations for each model are provided in the SI. This four-algorithm suite was intentionally designed to span instance-based (*k*NN), kernel-based (SVR), and ensemble-based (RF, XGBoost) learning paradigms, enabling systematic identification of the learning strategy most suited to each target's underlying transport mechanism. Detailed mathematical formulations and implementation specifics for each model are reported in the SI (Section S2).

### 3.3 Model optimization and validation strategy

Model hyperparameters were optimized using Bayesian optimization to efficiently explore high-dimensional parameter spaces while minimizing computational cost.^37–42^ The optimization process employed 50 iterations for each model and target variable, coupled with three-fold cross-validation on the training set. Mean squared error (MSE) served as the objective function during the optimization.

Model performance was assessed exclusively on the unseen test dataset using multiple metrics, including the coefficient of determination (*R*^2^), mean squared error (MSE), root mean squared error (RMSE), and mean absolute error (MAE). Residual diagnostics were applied to evaluate error structure and detect systematic deviations.^43^ Hyperparameter search spaces, optimized parameter values, and further validation diagnostics are reported in the SI.

## 4 Results and discussion

### 4.1 Membrane characterization and data collection

**Fig. 5:**
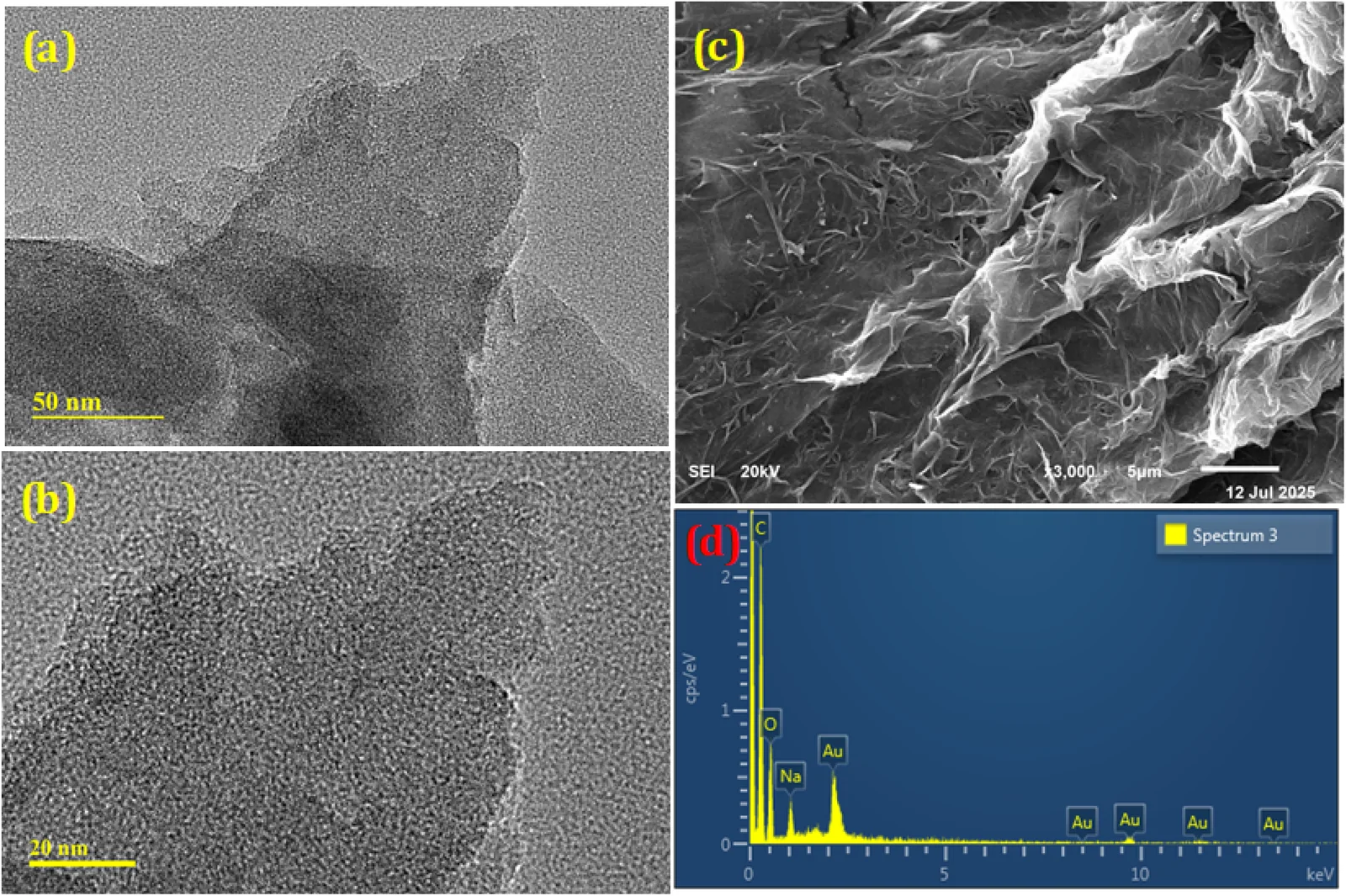
TEM image with a 50 nm scale bar (a) and TEM with 20 nm scale (b), SEM image (c), and EDX spectrum (d) of GO nanosheets prepared from waste graphite. Au peaks originate from gold sputter coating during the analysis.

The morphology of the obtained GO was confirmed by transmission electron microscopy (TEM), scanning electron microscopy-energy dispersive X-ray spectroscopy (SEM/EDX), Fig. 5. The TEM image shows ultrathin, semi-transparent GO nanosheets with pronounced wrinkles and folds, which are characteristic of GO.^44–46^ SEM images reveal crumpled and layered sheet-like morphology, characteristic of GO. EDX spectrum shows the presence of carbon and oxygen elements, confirming the oxidation of graphite into GO.^47^

**Fig. 6:**
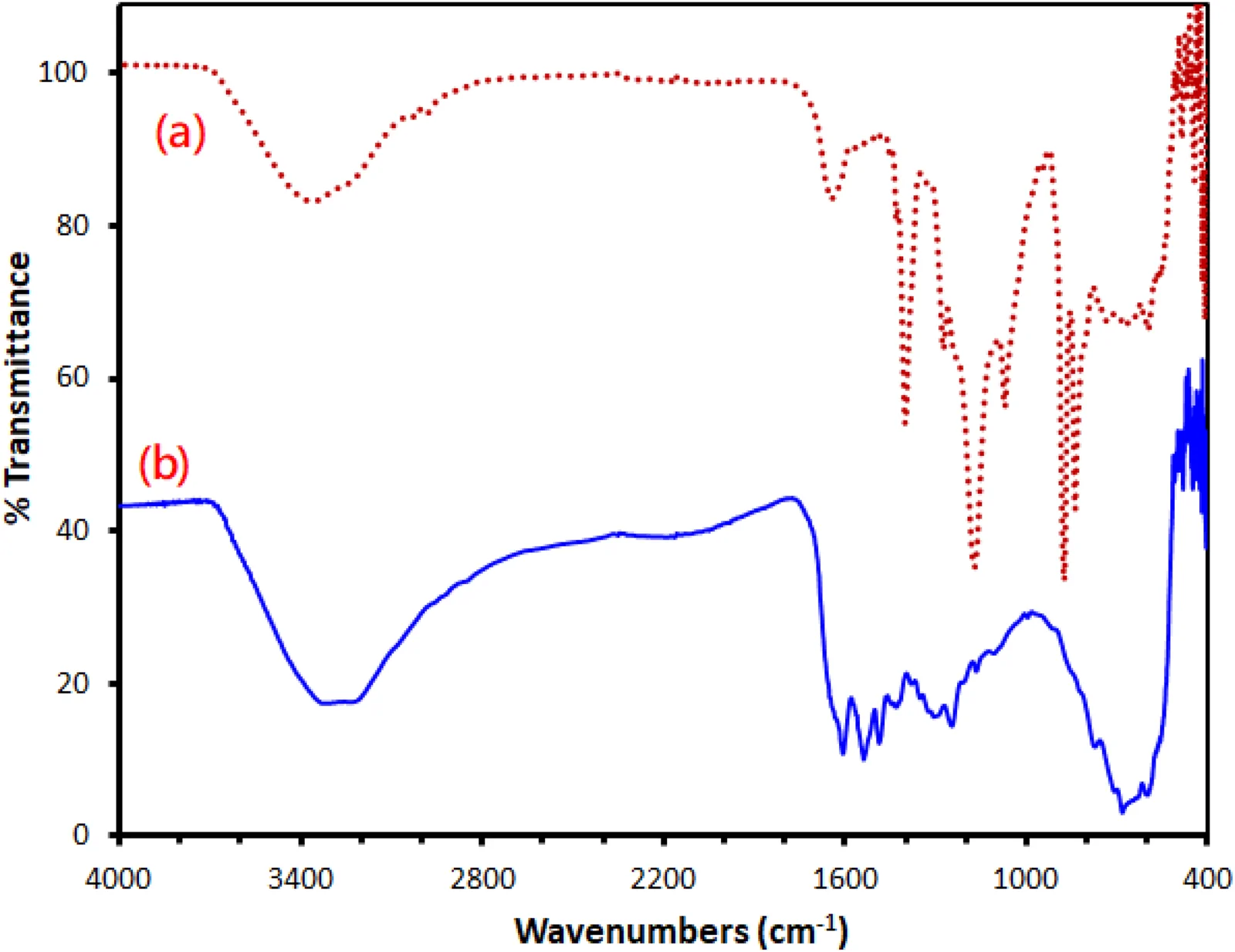
(a) FTIR spectrum of the functionalized GO, (b) FTIR spectrum of the developed GO-modified membranes.

The FTIR spectra confirm the successful modification of GO, Fig. 6. The IR spectrum of GO displays a broad band at ∼3200–3500 cm^−1^, which is attributed to O–H stretching, consistent with hydroxyl and carboxyl groups on the GO. The band at around 1650 cm^−1^ is ascribed to the C=O stretching. The additional bands in the 1200–1400 cm^−1^ region can be ascribed to C–N and C–O vibrations, confirming the success of functionalization.^48^ IR spectrum of the GO-modified membranes, the characteristic polyamide bands are observed at 3400 cm^−1^ (N–H stretching) and at 1670 cm^−1^ (C=O stretching), indicating possible formation of the polyamide layer.^49^ Bands in the 1000–1300 cm^−1^ region can be assigned to C–N stretching, confirming the integration of modified GO within the polymeric matrix.^50^

**Fig. 7:**
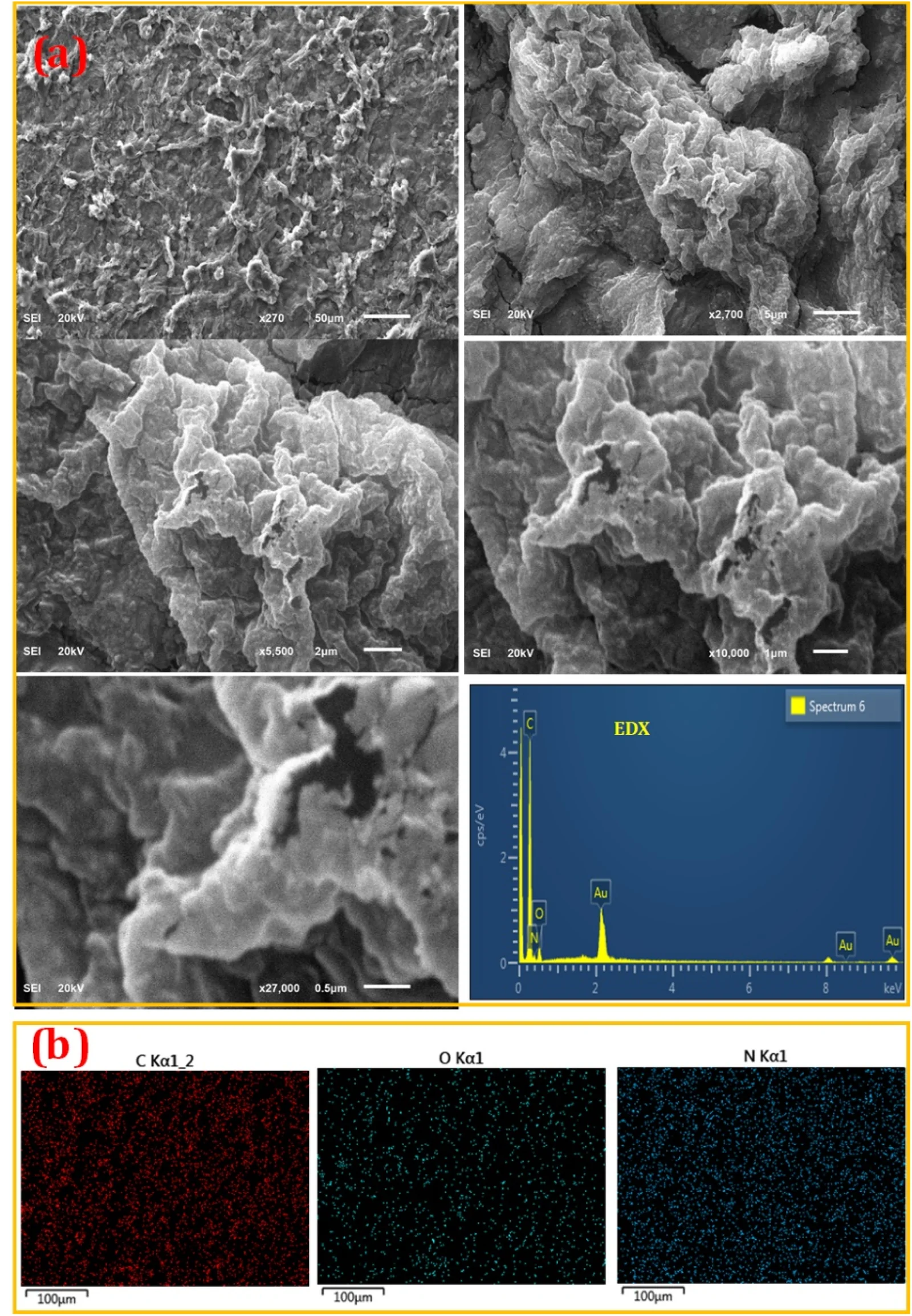
(a) SEM images of the prepared membranes with various magnification scales, with the corresponding EDX spectrum, and the elemental mapping of C, O, and N across the membranes (b).

The SEM/EDX analysis was performed on the prepared membranes, Fig. 7. SEM images display a wrinkled surface composed of crumpled GO-based nanosheets embedded within the polyamide matrix. The folded nanosheet-like morphology enhances the surface area and provides additional permeate pathways, which can enhance pollutant transport and membrane separation performance.

The presence of nitrogen, in addition to carbon and oxygen in the EDX spectrum, confirms that the modified GO was embedded within the membrane matrix. Thus, it verifies the polyamide layer formation on the membrane support.^7^ As shown in the elemental mapping images, the membrane surface displays a uniform distribution of N, and O elements. This indicates the homogeneous dispersion of GO within the polyamide layer without substantial aggregation, maintaining structural integrity and enhancing the flux properties.

### 4.2 Baseline statistical and linear modeling analysis

**Fig. 8:**
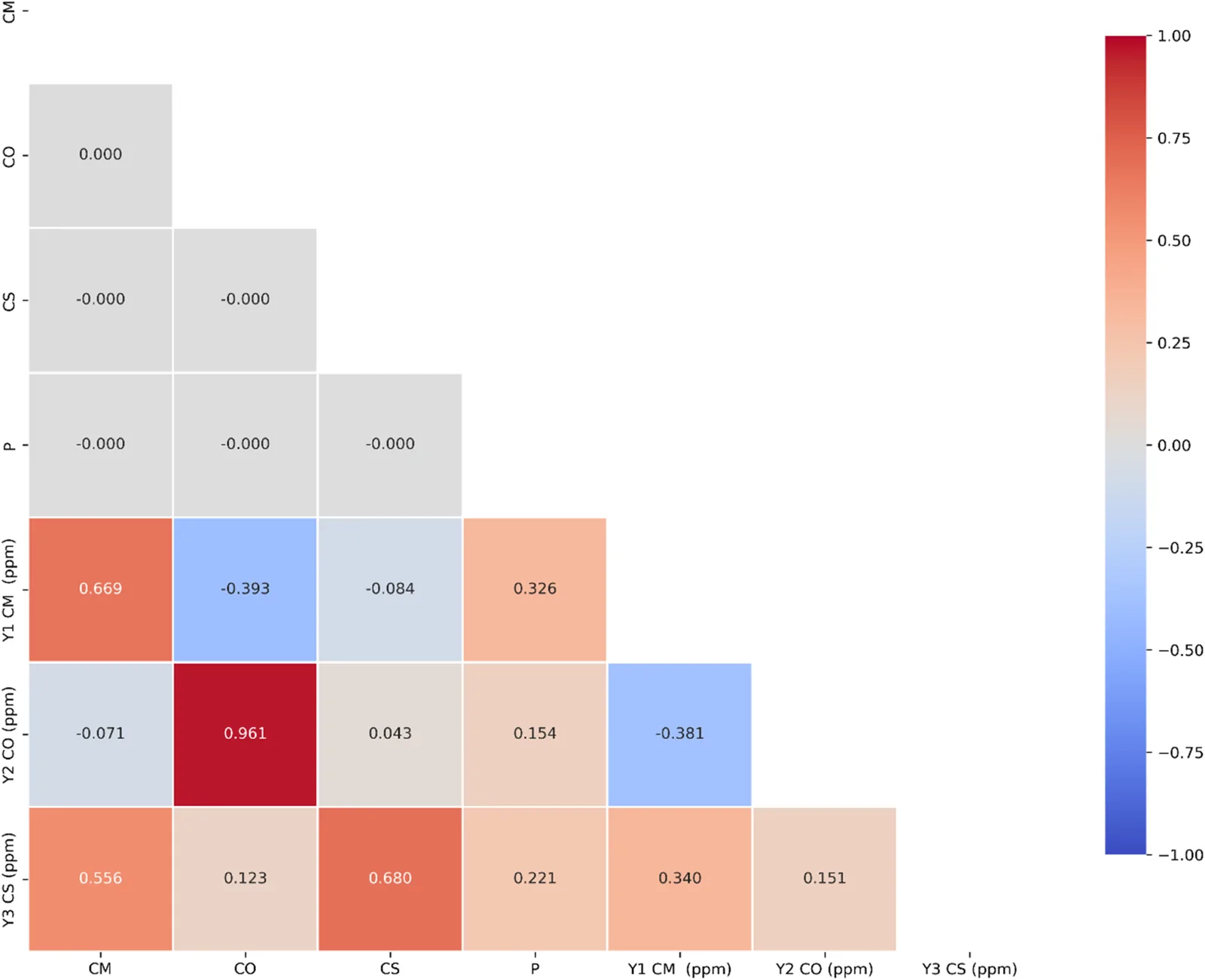
Pearson correlation heatmap with linear relationships between input variables (CM, CO, CS, P) and output responses (Y1, Y2, Y3).

**Fig. 9:**
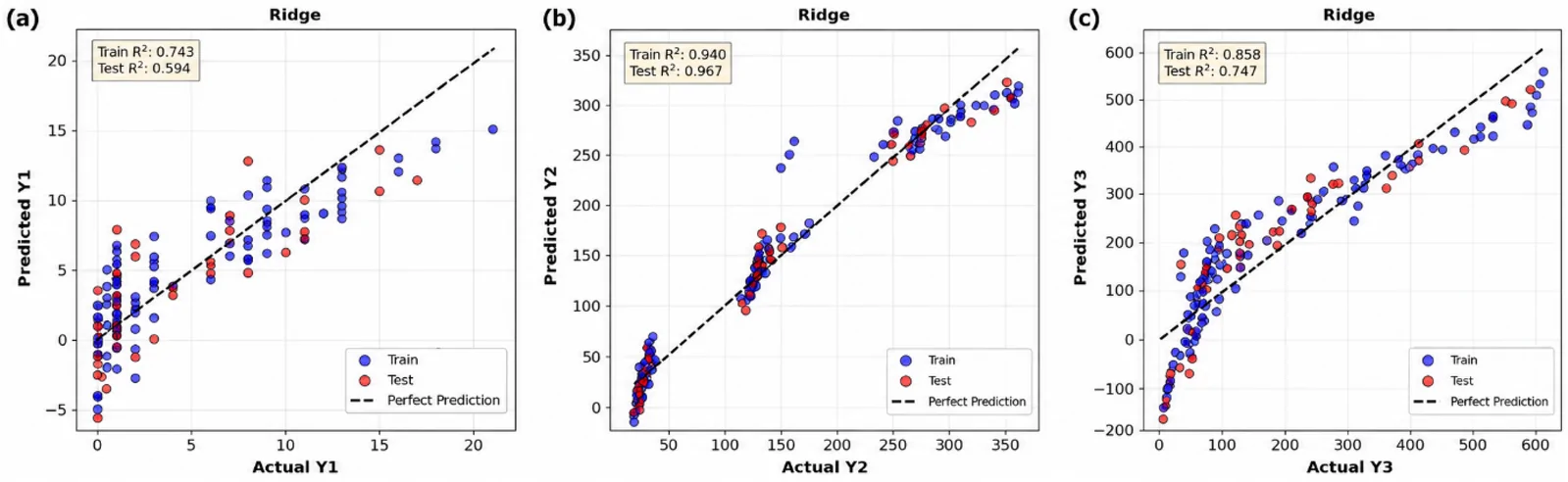
Optimized ridge regression predictions *versus* experimental values for (a) metal-ion permeate concentration (Y1), (b) organic-pollutant permeate concentration (Y2), and (c) salt permeate concentration (Y3).

Baseline statistical and linear regression analyses were performed prior to ML modeling to evaluate whether membrane performance could be adequately described using simple linear relationships, Fig. 8. Pearson correlation analysis displayed moderate correlations between feed concentration and the corresponding permeate metal-ion concentration (CM–Y1, *r* ≈ 0.67) and salt-ion concentration (CS–Y3, *r* ≈ 0.68). A strong correlation was observed between organic pollutant concentration and permeate concentration (CO–Y2, *r* ≈ 0.96). Pressure showed weaker correlations with all output variables, indicating a secondary but non-dominant effect on separation performance. Correlation analysis provides a first-order indication of linear dependence, but it does not explain nonlinear interactions or conditional dependencies between variables.^34^ The distribution and variability of both input and output variables indicate discrete experimental levels, skewed response distributions, and clustered data patterns rather than continuous linear trends, Fig. S1–S4. In particular, Y1 and Y3 show increased variance at higher concentration levels, suggesting heteroscedastic behavior that challenges linear model assumptions. Ridge regression was used as the linear baseline model for each target variable to quantify predictive capability. The predicted *versus* experimental values for Y1, Y2, and Y3 using the optimized linear regression model are shown in Fig. 9. For Y1, the linear model achieved a training *R*^2^ of 0.74 and a test *R*^2^ of 0.59, indicating limited generalization and substantial unexplained variance. Organic pollutant permeate concentration (Y2) was described reasonably well by the linear model, with training and test *R*^2^ values of 0.95 and 0.97, indicating a strong linear correlation. The linear regression model for salt-ion concentration (Y3) demonstrated moderate predictive performance, achieving an *R*^2^ of 0.86 on the training set and 0.75 on the test set. These results indicate that a linear model is insufficient to capture the complex relationships between the input variables and the target outputs. Therefore, nonlinear regression models are required to improve predictive performance.

Analysis of the predicted *versus* actual plots shows systematic deviations from the ideal parity line, mainly at higher concentrations, and increasing prediction spread with response magnitude. These deviations are consistent with non-uniform variance and nonlinear response patterns, Fig. S1–S4. The residual structures imply non-random error distributions, which are commonly used diagnostic markers of regression model misspecification.^43^

### 4.3 ML prediction of nanofiltration performance

**Fig. 10:**
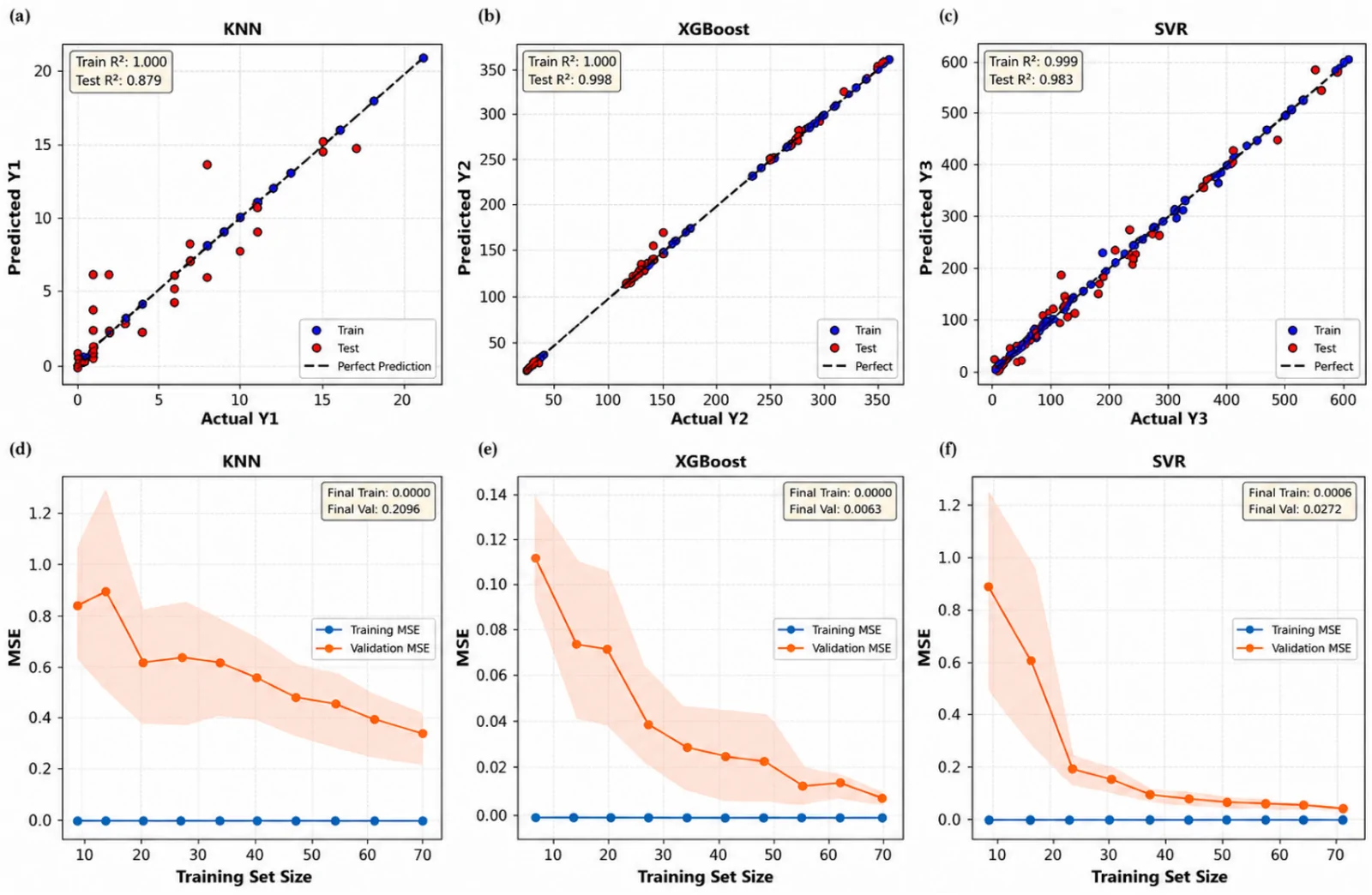
Predicted *versus* experimental values and learning curves for the best-performing machine-learning models: (a and d) *k*NN for metal-ion permeate concentration (Y1), (b and e) XGBoost for organic-pollutant permeate concentration (Y2), and (c and f) SVR for salt permeate concentration (Y3).

The *k*-nearest neighbors (*k*NN) regressor showed the best prediction accuracy for Y1, with an *R*^2^ of 0.879 and a test RMSE of 1.65 ppm. The predicted values closely followed the parity line for both the training and test datasets, indicating good agreement between the predicted and experimental values, Fig. 10a. The learning plot displays stable convergence and limited overfitting, as shown in Fig. 10d. In contrast, the Random Forest, SVR, and XGBoost models exhibited lower predictive performance, as shown in Fig. S5 and S6. The robust performance of *k*NN suggests that metal-ion concentration is controlled by localized response behavior within the operating parameter space, which is more effectively captured by instance-based learning than by global regression models.

Organic pollutant permeates concentration (Y2) showed the highest predictive accuracy, with XGBoost reaching an *R*^2^ of 0.998 and a test RMSE of 4.81 ppm. Predicted values followed the parity line for both the training and test datasets, indicating that the model was not overfitted and exhibited good generalization performance, Fig. 10b. Furthermore, the learning curve indicates stable model convergence and minimal overfitting, as shown in Fig. 10e. The high performance for Y2 aligns with the strong linear correlation between feed concentration and permeate concentration observed in the baseline analysis. Nevertheless, comparison with alternative models indicates that nonlinear learning remains beneficial. While Random Forest achieved comparable accuracy, SVR and linear regression displayed increased prediction errors, particularly at higher concentration levels. Comparisons between predicted and experimental values, along with residual distributions for the assessed models, are shown in Fig. S7 and S8. XGBoost efficiently captures both main concentration effects and secondary nonlinear interactions affecting the permeation of organic contaminants.

With a test *R*^2^ of 0.983 and a test RMSE of 20.9 ppm, the support vector regression (SVR) model accurately predicted the permeate concentration of salts (Y3). For both training and test datasets, SVR predictions clearly followed the parity line, demonstrating robust generalization, Fig. 10c. The corresponding learning plot shows stable convergence with a slight difference between the training and validation errors, Fig. 10f. SVR consistently outperformed *k*NN, Random Forest, and XGBoost in both error magnitude and test accuracy. Although ensemble tree-based models were able to capture the general trend, they displayed greater residual spread, primarily at high salt concentrations, Fig. S9 and S10. The superior performance of SVR indicates that salt passage is controlled by smoother, continuous response behavior, which is more effectively captured by kernel-based regression than by instance-based or tree-based models.

### 4.4 Model comparison and robustness analysis

Across all targets, no single model consistently outperformed the others, highlighting the importance of target-specific model selection. For Y1, *k*NN attained the highest test accuracy (*R*^2^ = 0.879, RMSE = 1.65 ppm), outperforming all other evaluated models, which displayed higher prediction errors. Organic pollutant permeate concentration (Y2) showed the strongest overall predictability, with XGBoost providing a balance between variance explanation (*R*^2^ = 0.998) and error minimization (RMSE = 4.81 ppm). *k*NN showed some overfitting behavior, characterized by perfect training performance and degraded test accuracy. Y3 was the most challenging prediction task, where SVR achieved strong generalization (*R*^2^ = 0.983, RMSE = 20.9 ppm), outperforming tree-based and instance-based models.

**Fig. 11:**
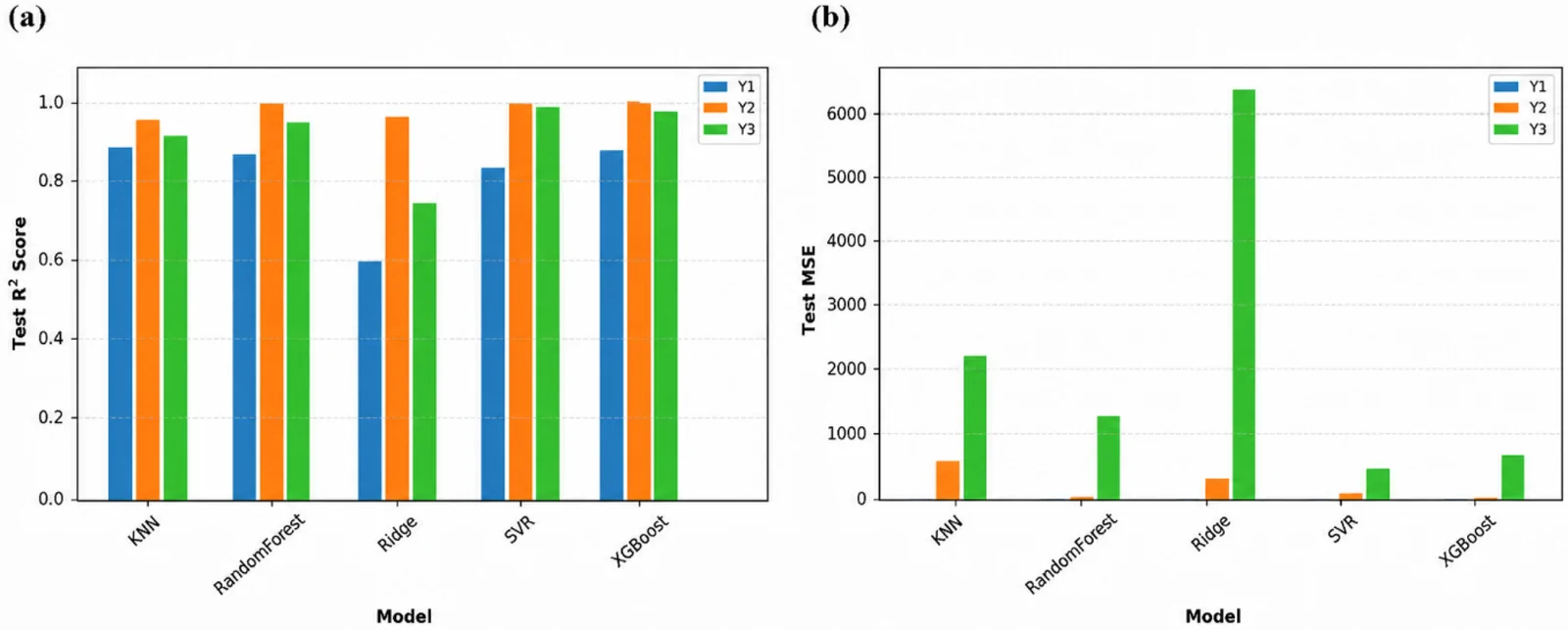
Comparative test-set performance of the investigated machine-learning models for metal-ion permeate concentration (Y1), organic-pollutant permeate concentration (Y2), and salt permeate concentration (Y3), evaluated using (a) *R*^2^ and (b) MSE.

The selected best-performing models maintained robust generalization, whereas models exhibiting near-perfect training performance without corresponding test accuracy highlighted the risk of overfitting, Fig. 11. These findings confirm that reliable NF performance prediction requires adaptive, target-specific model selection rather than reliance on a single universal algorithm. XGBoost is recommended for integration into the portable unit's predictive interface owing to its superior generalization for organic contaminant prediction, computational efficiency during inference, and robustness across the full range of tested operating conditions, attributes that make it well-suited for real-time process guidance under variable feed compositions.

## 5 Design of portable wastewater treatment unit

**Fig. 12:**
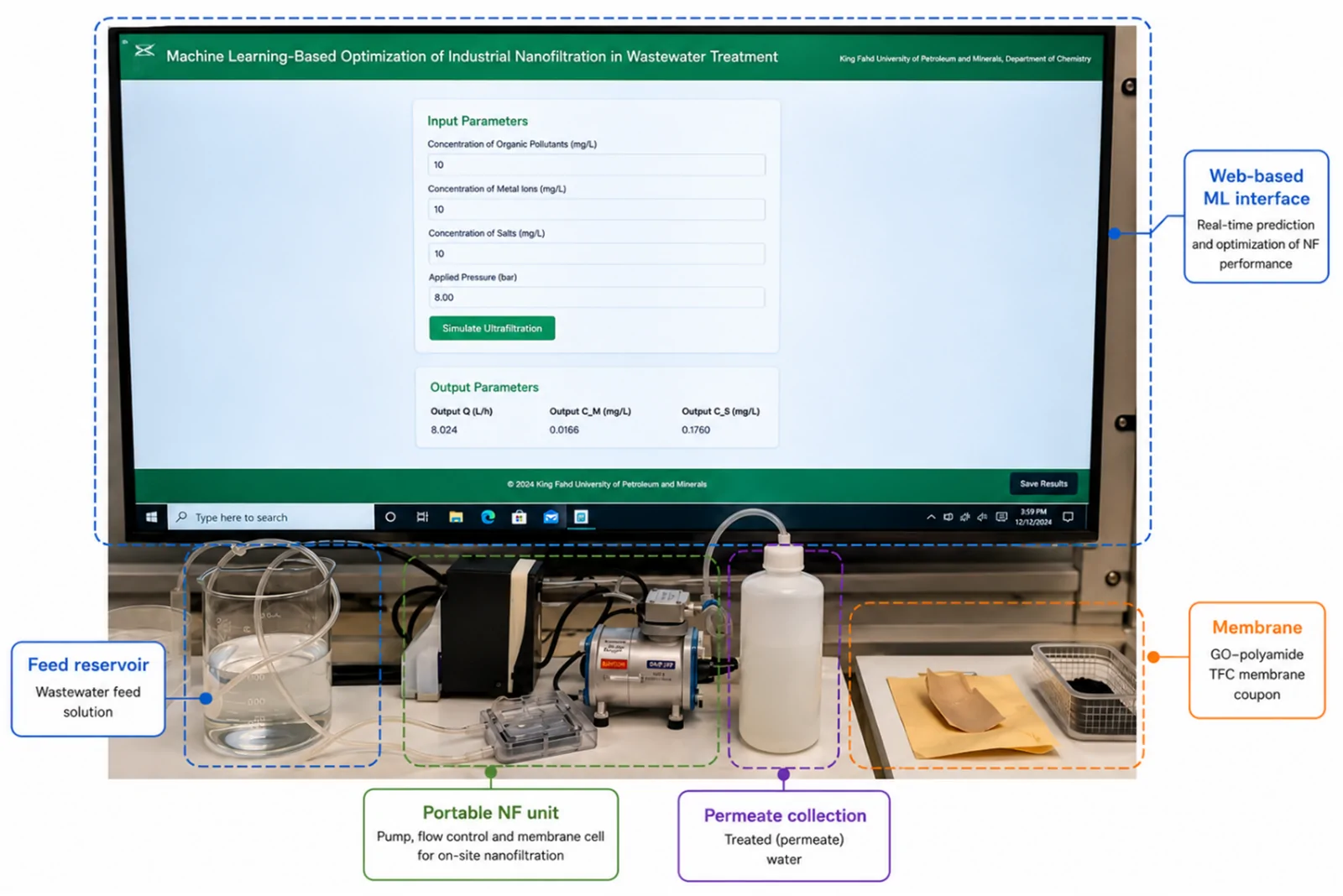
The developed water treatment unit with interface control (upper panel) and the design of the portable wastewater treatment device (lower panel).

Following validation of the ML-NF integration, a portable bench-top NF unit was designed and assembled to deploy the ML optimization framework in a real-world demonstration, Fig. 12. The validated framework was integrated into the mobile unit, which was equipped with a web-based predictive interface that provides operators with actionable guidance under variable feedwater conditions. The unit consists of the GO-modified TFC membrane, a compact diaphragm pump, a flat-sheet membrane cell module, a feed reservoir, and a permeate collection vessel mounted on a portable workstation. The system operates in crossflow mode, and the feed solution is introduced from the reservoir into the membrane module, where separation takes place across the membrane. The permeate is collected downstream, and the retentate is recirculated. All fluidic connections use chemically resistant tubing compatible with organic pollutants, metal ions, and inorganic salts.

The developed unit combines cost-effective nanofiltration membranes with a web-based machine learning interface that is accessible from any network-enabled device. The interface enables both system operation and real-time prediction of membrane performance and contaminant rejection. Users can enter the operating conditions, including the feed concentrations of organic pollutants (CO), metal ions (CM), salt ions (CS), and the applied pressure, after which the system instantly predicts the permeate concentrations of the target contaminants. These features enable pre-evaluation of operating conditions before physical operation, reducing chemical consumption, energy use, and fouling risks. The deployed models, *k*NN for metal-ion removal, XGBoost for organic pollutant concentration, and SVR for salt passage, function as backend inference engines that can be updated independently of the hardware.

The developed device is suitable for remote locations, small-scale industrial facilities, and on-site water treatment scenarios where conventional infrastructure may be limited. As a limitation, the current system operates at a laboratory demonstration scale (Technology Readiness Level, TRL 3–5). However, scale-up to an industrial level would require further engineering optimization, including membrane area expansion, module design, and long-term stability evaluation.

## 6 Preliminary techno-economic assessment

A preliminary techno-economic evaluation was performed to assess the practical feasibility and scalability of the developed portable unit. Capital expenditure (CAPEX) and operational expenditure (OPEX) can be assessed to evaluate deployment potential. Capital costs are mainly governed by membrane fabrication. Interestingly, the use of waste graphite as a GO feedstock significantly decreases raw material expenditure relative to commercially sourced materials. The remaining components, including the membrane module, tubing, and diaphragm pump, are low-cost and commercially available. This enables the unit fabrication at an estimated laboratory-scale cost below $800–1000, depending on component specifications.

Additionally, the developed unit operates at moderately low pressure, which allows the use of low-power pumps with an estimated energy consumption of 0.2–0.5 kWh m^−3^. This is significantly lower than pressure-driven processes such as reverse osmosis (1.5–3 kWh m^−3^). The energy reduction demand contributes to lower operating costs and enhanced sustainability.

The ML integration predictive interface offers indirect economic benefits through trial-and-error experimentations, mitigating membrane fouling, and minimizing chemical usage through optimized operating conditions. Such predictive capability extends membrane lifespan and minimizes downtime, which results in cumulative cost savings over long-term operation. Thus, the developed unit offers a cost-competitive and energy-efficient solution for decentralized water reuse, supporting sustainable clean water production through the closed-loop integration of ML-guided process optimization and GO-membranes.

## 7 Conclusion

In this work, GO-modified polyamide TFC membranes were synthesized, characterized, and integrated with predictive ML models to optimize nanofiltration performance for industrial wastewater treatment. In the first stage, imidazole-functionalized GO was incorporated into polyamide TFC membranes, and the resulting composites were characterized by FTIR, SEM, TEM, EDX, and elemental mapping, confirming successful GO functionalization and its homogeneous dispersion within the polyamide matrix. The membranes were evaluated in a crossflow NF system, generating a 135-experiment dataset covering systematic variations in transmembrane pressure and feed composition for three pollutant classes. Target-specific ML models were developed: *k*NN for metal-ion prediction, XGBoost for organic pollutant prediction, and SVR for salt passage prediction. The target-specific model selection strategy demonstrated that instance-based, ensemble, and kernel-based learning methods each optimally capture the distinct transport mechanisms governing NF rejection of different contaminant classes. This combined experimental-ML approach transforms NF operation from empirical trial-and-error toward data-informed process intensification, establishing a scalable and practical pathway for advanced NF membrane deployment in variable industrial water reuse scenarios. Future work should extend validation to larger-scale modules, pilot-level operation, and long-term fouling studies.

## Author contributions

M. T. B., M. M. B. and T. A. S. have contributed to the synthetic work and data analysis. M. T. B.: methodology, investigation, experimental data collection, machine-learning model development and validation, data analysis, visualization, manuscript writing, and editing. M. M. B.: material synthesis, membrane fabrication, nanofiltration experiments, and experimental data collection. T. A. S.: conceptualization, supervision, resources, manuscript review, and editing.

## Conflicts of interest

There are no conflicts to declare.

## Supplementary Material

RA-OLF-D6RA05062J-s001

## Data Availability

All data supporting this article have been included in the main text and the supplementary information (SI). Supplementary information: detailed descriptions of ML models and hyperparameter optimization; definitions of input and output variables (Table S1); additional statistical analyses including data distribution plots (Fig. S1–S4); parity plots and residual analyses for all evaluated models (Fig. S5–S10); extended model validation results; and supplementary methodological details. See DOI: https://doi.org/10.1039/d6ra05062j.
