# Supplementary material for: Sustainable nanofiltration of industrial wastewater using waste-derived-graphene oxide-modified polyamide membranes: machine learning toward a smart portable water treatment unit
Source: RSC Adv. 2026 Sep 3;16(43):52682–95. doi: 10.1039/d6ra05062j (PMC13539501; doi:10.1039/d6ra05062j)
Supplement: Supplementary file 1 [file RA-016-D6RA05062J-s001.pdf]

Supplementary Information (SI) for RSC Advances.
This journal is © The Royal Society of Chemistry 2026

Sustainable Nanofiltration of Industrial Wastewater Using Waste-Derived-Graphene Oxide-Modified Polyamide Membranes: Machine Learning toward a Smart Portable Water Treatment Unit

Montassar T. Bouzidi[1], Mariem M. BenSlimene[1], Tawfik A. Saleh[1,2*]

[1] Department of Chemistry, King Fahd University of Petroleum & Minerals, Dhahran 31261, Saudi Arabia

[2] Interdisciplinary Research Center for Advanced Materials, King Fahd University of Petroleum & Minerals, Dhahran, 31261, Saudi Arabia

*Correspondence to:

E-mail address: tawfikas@hotmail.com; tawfik@kfupm.edu.sa

Table S1: Input and Output Variables

Category	Variable	Description
Inputs	CM (ppm)	Concentration of metal ions in feedwater.
	CO (ppm)	Concentration of organic pollutants in feedwater.
	CS (ppm)	Concentration of salts in feedwater.
	Pressure	*Applied pressure during nanofiltration.*
Outputs	Y1 (ppm)	Permeate concentration of metal ions.
	Y2 (ppm)	Permeate concentration of organic pollutants.
	Y3 (ppm)	Permeate concentration of salts.

S2. Machine Learning Models and Optimization Framework

S2.1. Linear Regression (Baseline Model)

Optimized linear regression was used as a baseline model to assess linear relationships between input variables and membrane performance. The model considers a linear mapping between predictors and the response variable:

$$\hat{y} = \beta_0 + \sum_{i=1}^{n} \beta_i x_i$$

where $\hat{y}$ is the predicted output, x_i are the input features, and β_i are regression coefficients estimated by minimizing the residual sum of squares:

$$\min_{\beta} \sum_{j=1}^{m} \left(y_j - \hat{y}_j \right)^2$$

This model provides a reference point for evaluating the benefits of nonlinear learning algorithms.

S2.2. K-Nearest Neighbors (KNN)

kxK-nearest neighbors regression predicts the target value based on the average response of the closest samples in feature space. For a query point, the prediction is given by:

$$\hat{y}(x) = \frac{1}{k} \sum_{i \in N_k(x)} y_i$$

$N_k(x)kx$where the nearest-neighbor set is defined according to a distance metric (Euclidean distance in this work). Distance-weighted averaging was applied to reduce sensitivity to local noise.

S2.3. Support Vector Regression (SVR)

εSupport vector regression constructs a function that deviates from the true targets by no more than a predefined margin while maintaining model flatness. The regression function is defined as:

$$f(x) = w^T \phi(x) + b$$

The optimization problem is formulated as:

$$\min_{w,b,\xi,\xi^*} \frac{1}{2} \| w \|^2 + C \sum_{i=1}^{m} \left(\xi_i + \xi_i^* \right)$$

subject to:

$$y_i - f(x_i) \leq \varepsilon + \xi_i$$
$$f(x_i) - y_i \leq \varepsilon + \xi_i^*$$

A radial basis function (RBF) kernel was used to capture nonlinear dependencies.

S2.4. Random Forest Regression

Random forest regression is an ensemble learning method that aggregates predictions from multiple decision trees trained on bootstrap samples of the dataset. The model prediction is given by:

$$\hat{y} = \frac{1}{T}\sum_{t=1}^{T} h_t(x)$$

$h_t(x)t$where each term represents the prediction of an individual tree. Feature randomness at each split reduces correlation between trees and improves generalization.

S2.5. Extreme Gradient Boosting (XGBoost)

XGBoost is a gradient boosting algorithm that builds trees sequentially to minimize a regularized objective function:

$$L = \sum_{i=1}^{m} l(y_i,\hat{y}_i) + \sum_{k=1}^{K} \Omega(f_k)$$

with the regularization term:

$$\Omega(f) = \gamma T + \frac{1}{2}\lambda \parallel w \parallel ^2$$

$Tw$where the terms define the number of leaves and the corresponding leaf weights. Second-order Taylor expansion of the loss function enables efficient optimization and improved convergence.

S2.6. Model Evaluation Metrics

Model performance was evaluated using multiple regression metrics:

- R^2Coefficient of determination (R^2)

$$R^2 = 1 - \frac{\Sigma(y_i - \hat{y}_i)^2}{\Sigma(y_i - \bar{y})^2}$$

- **Mean Squared Error (MSE)**

$$MSE = \frac{1}{m}\Sigma(y_i - \hat{y}_i)^2$$

- **Root Mean Squared Error (RMSE)**

$$RMSE = \sqrt{MSE}$$

- **Mean Absolute Error (MAE)**

$$MAE = \frac{1}{m}\Sigma|y_i - \hat{y}_i|$$

Residual diagnostics were used to evaluate error structure and detect systematic deviations.

S2.7. Bayesian Hyperparameter Optimization

$f(\theta)$Hyperparameter optimization was conducted using Bayesian optimization, which models the objective function as a probabilistic surrogate, typically a Gaussian process (GP):

$$f(\theta) \sim GP(\mu(\theta),k(\theta,\theta'))$$

$a(\theta)$An acquisition function balances exploration and exploitation to select the next evaluation point:

$$\theta^* = \underset{\theta}{argmax}\, a(\theta)$$

The expected improvement (EI) criterion was used in this study:

$$EI(\theta) = E\left[max\left(f_{best} - f(\theta), 0\right)\right]$$

This approach enables efficient exploration of high-dimensional hyperparameter spaces with a limited number of evaluations.

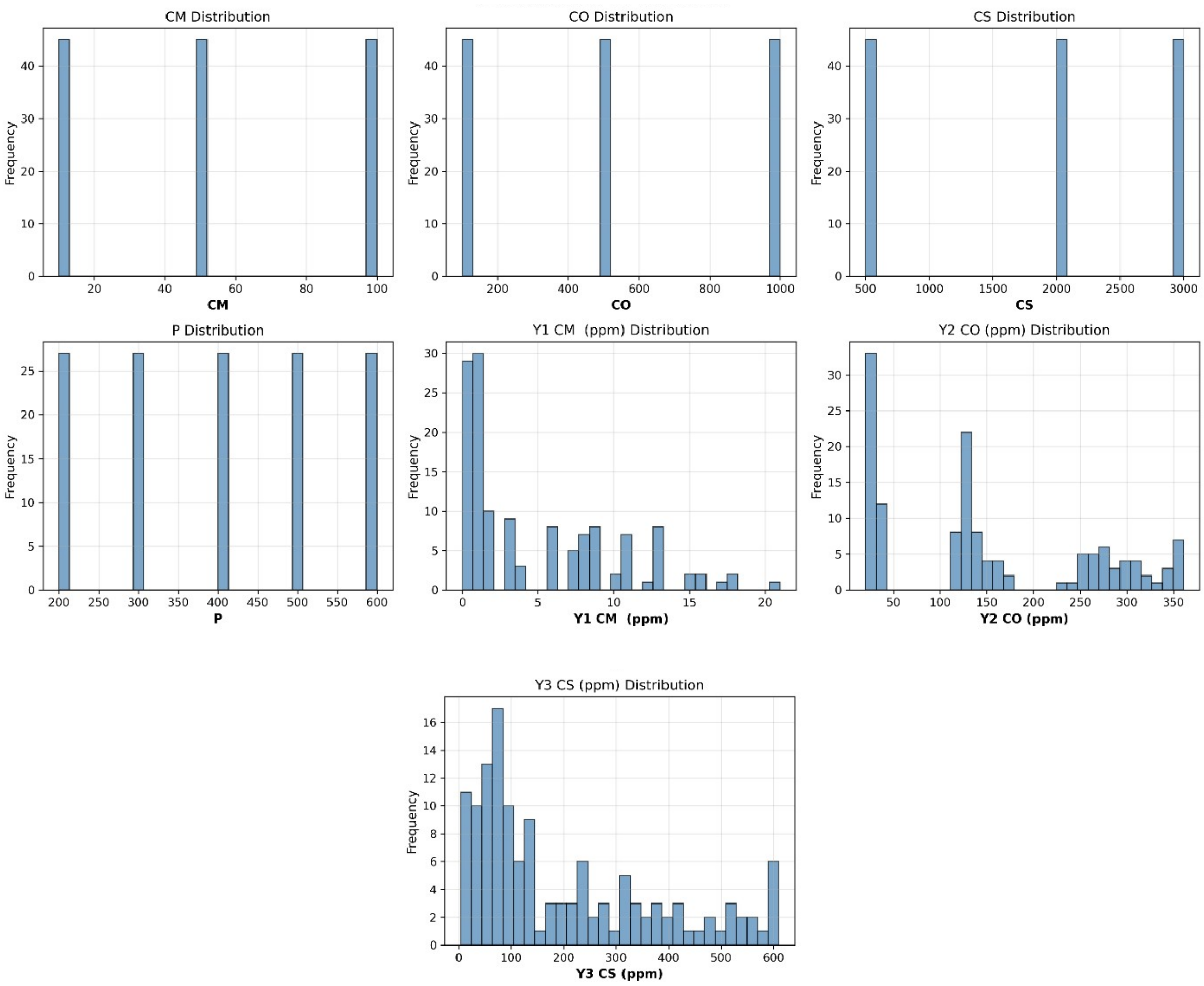

Figure S1. Distribution of input (CM, CO, CS, P) and output (Y1, Y2, Y3) variables across the experimental dataset.

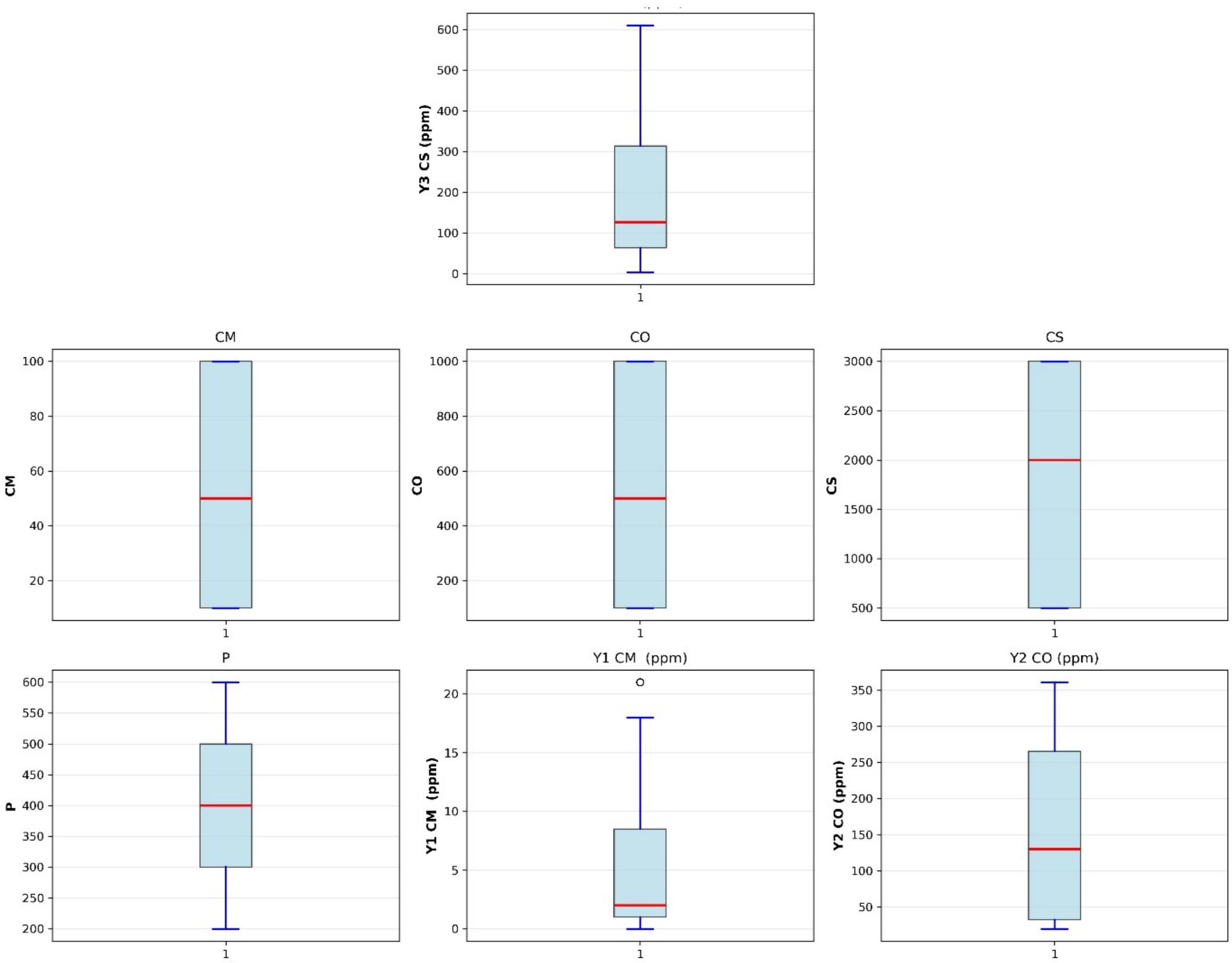

Figure S2. Box plots of input and output variables illustrating data spread and the absence of extreme outliers.

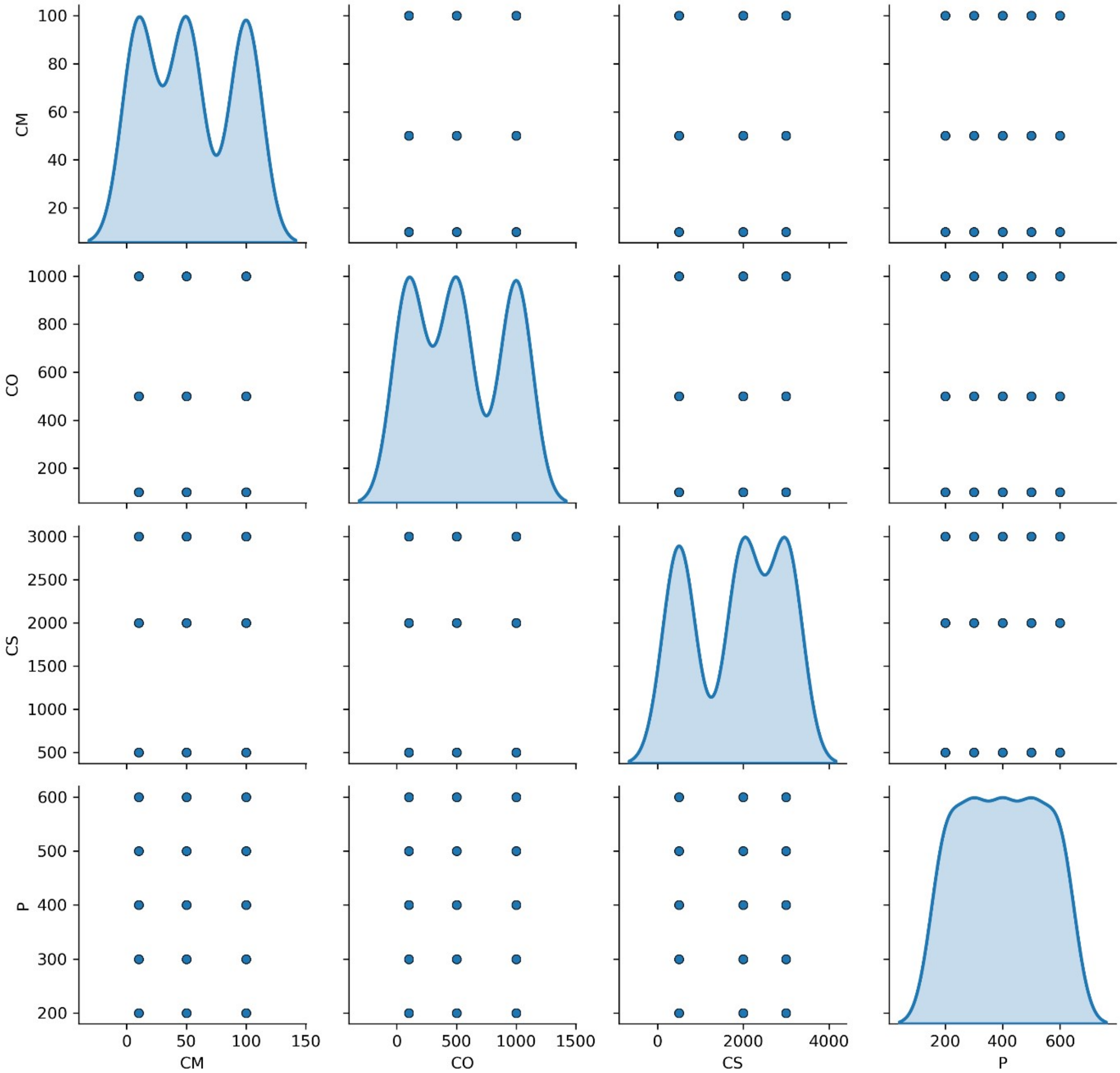

Figure S3. Pairwise scatter plots and marginal distributions showing relationships between input variables and membrane performance metrics.

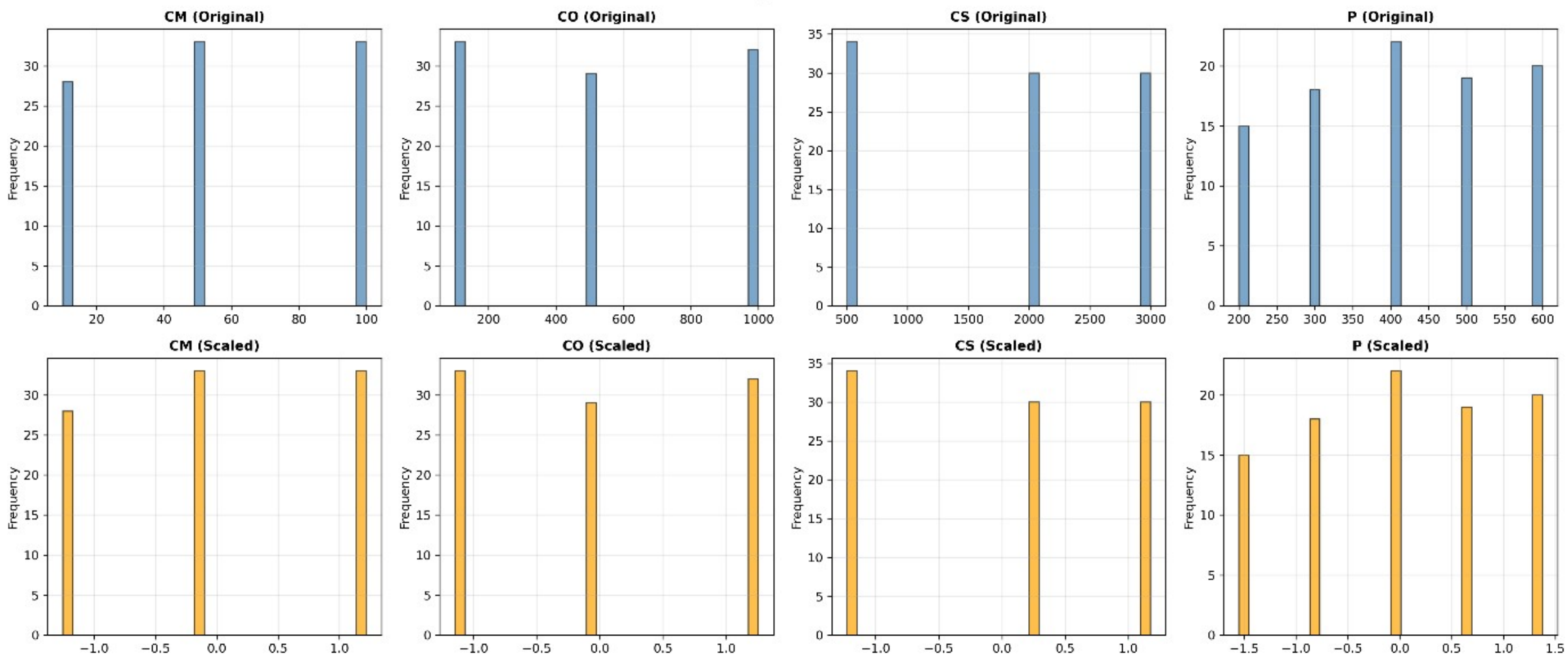

Figure S4. Feature scaling comparison before and after standardization for all input variables.

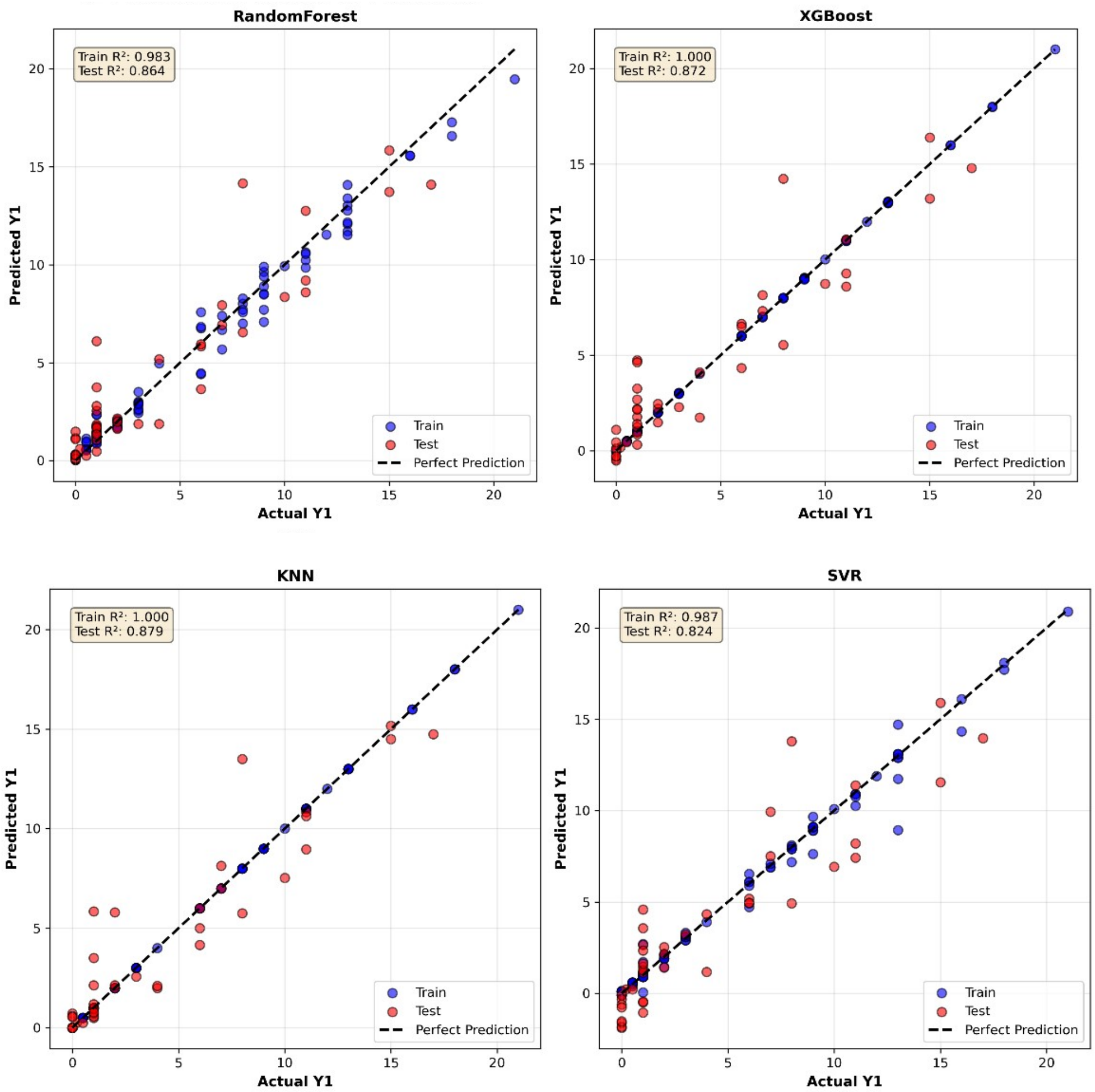

Figure S5. Predicted versus experimental values for Y1 obtained using Random Forest, XGBoost, KNN, and SVR models.

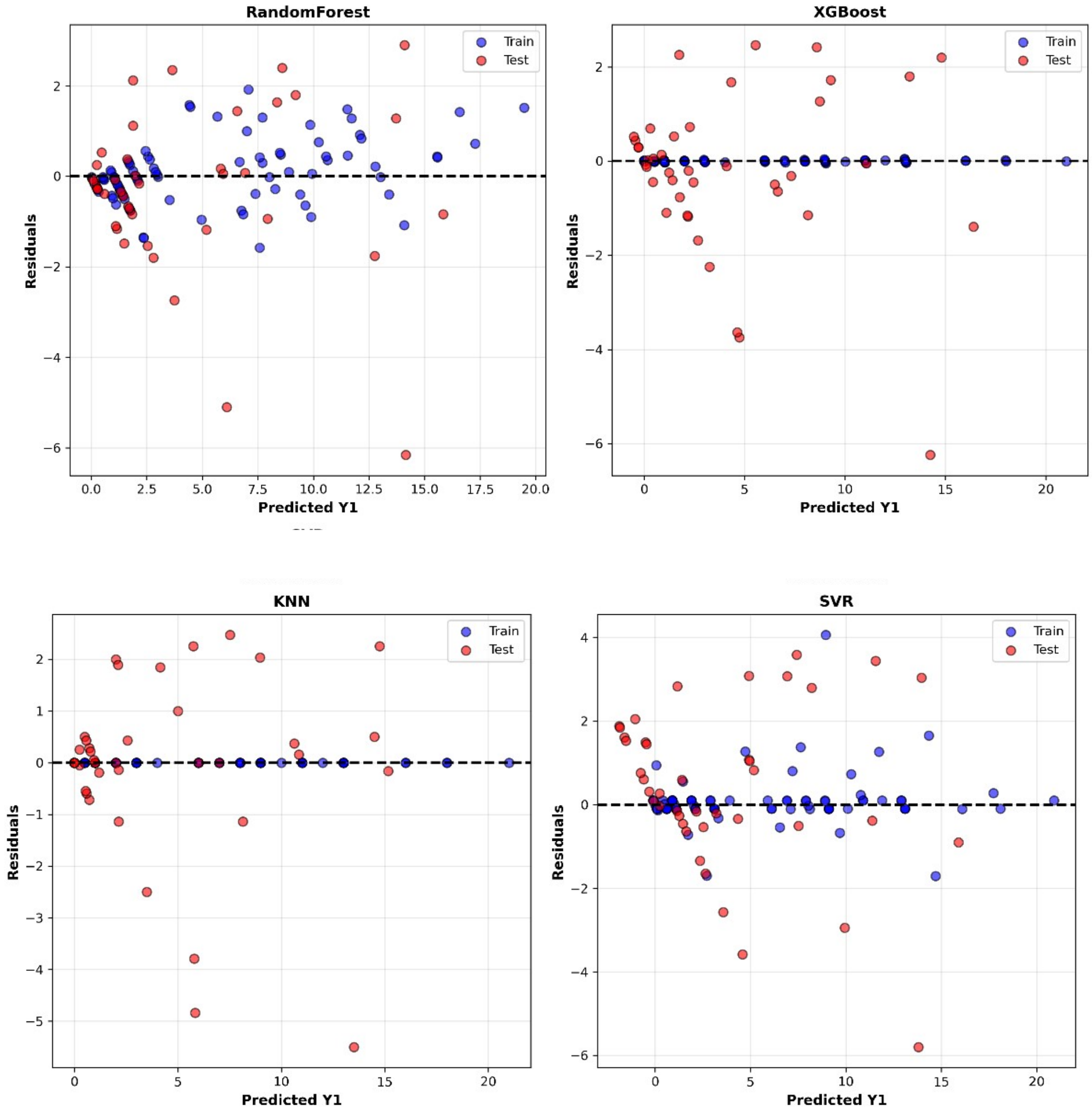

Figure S6. Residual distributions for Y1 predictions using Random Forest, XGBoost, KNN, and SVR models.

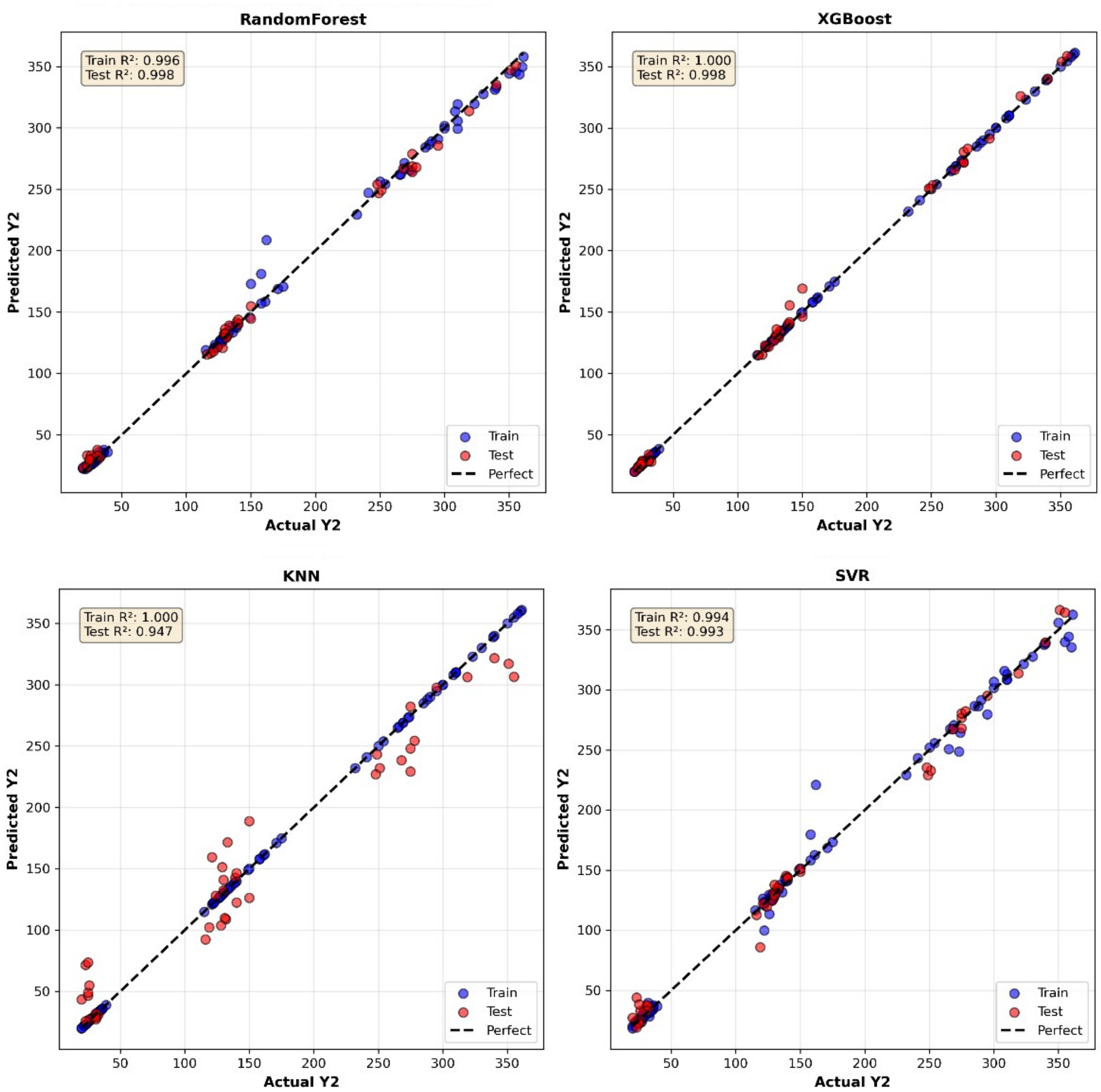

Figure S7. Predicted versus experimental values for Y2 obtained using Random Forest, XGBoost, KNN, and SVR models.

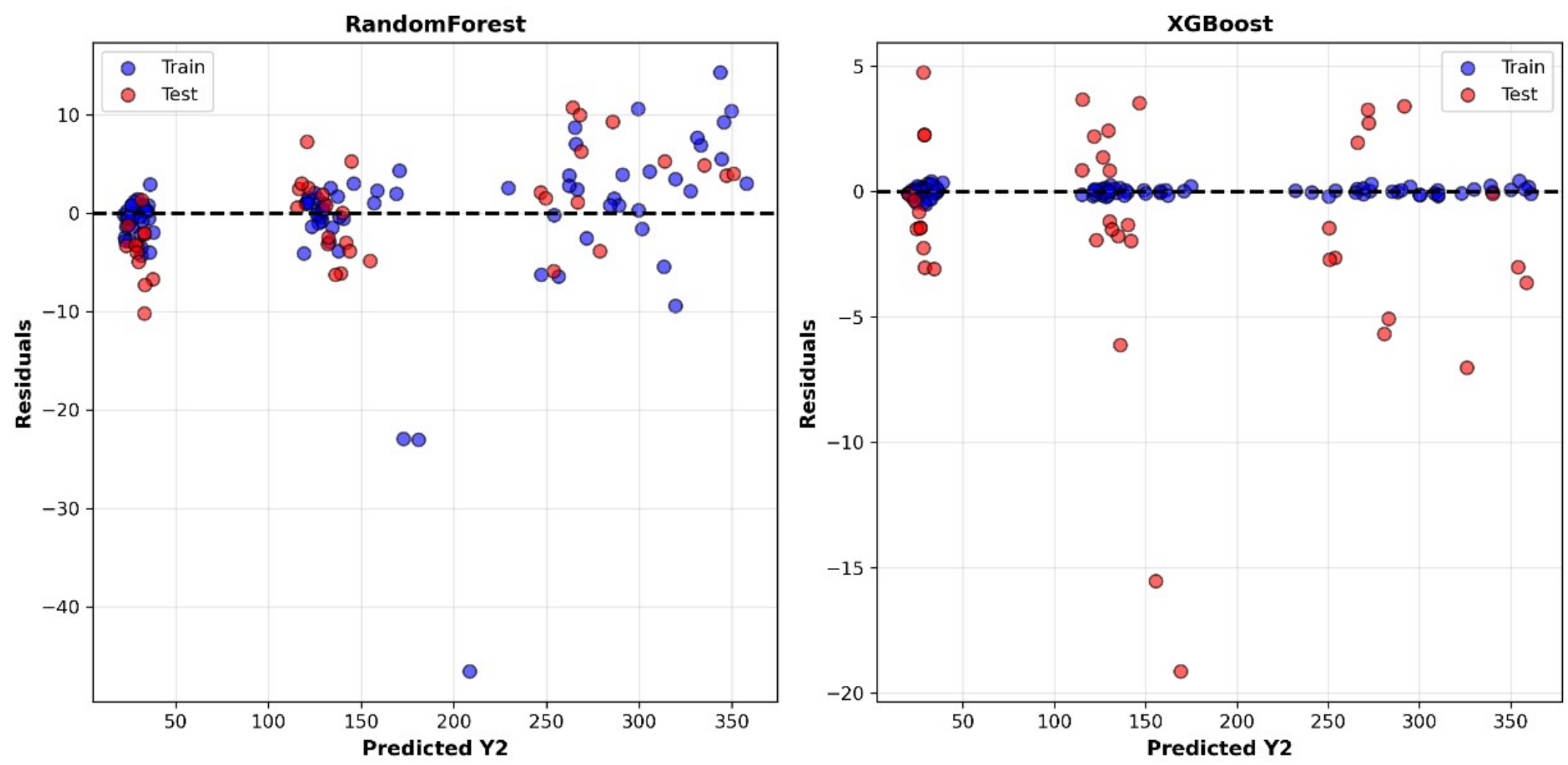

Figure S8. Residual distributions for Y2 predictions using Random Forest, XGBoost, KNN, and SVR models.

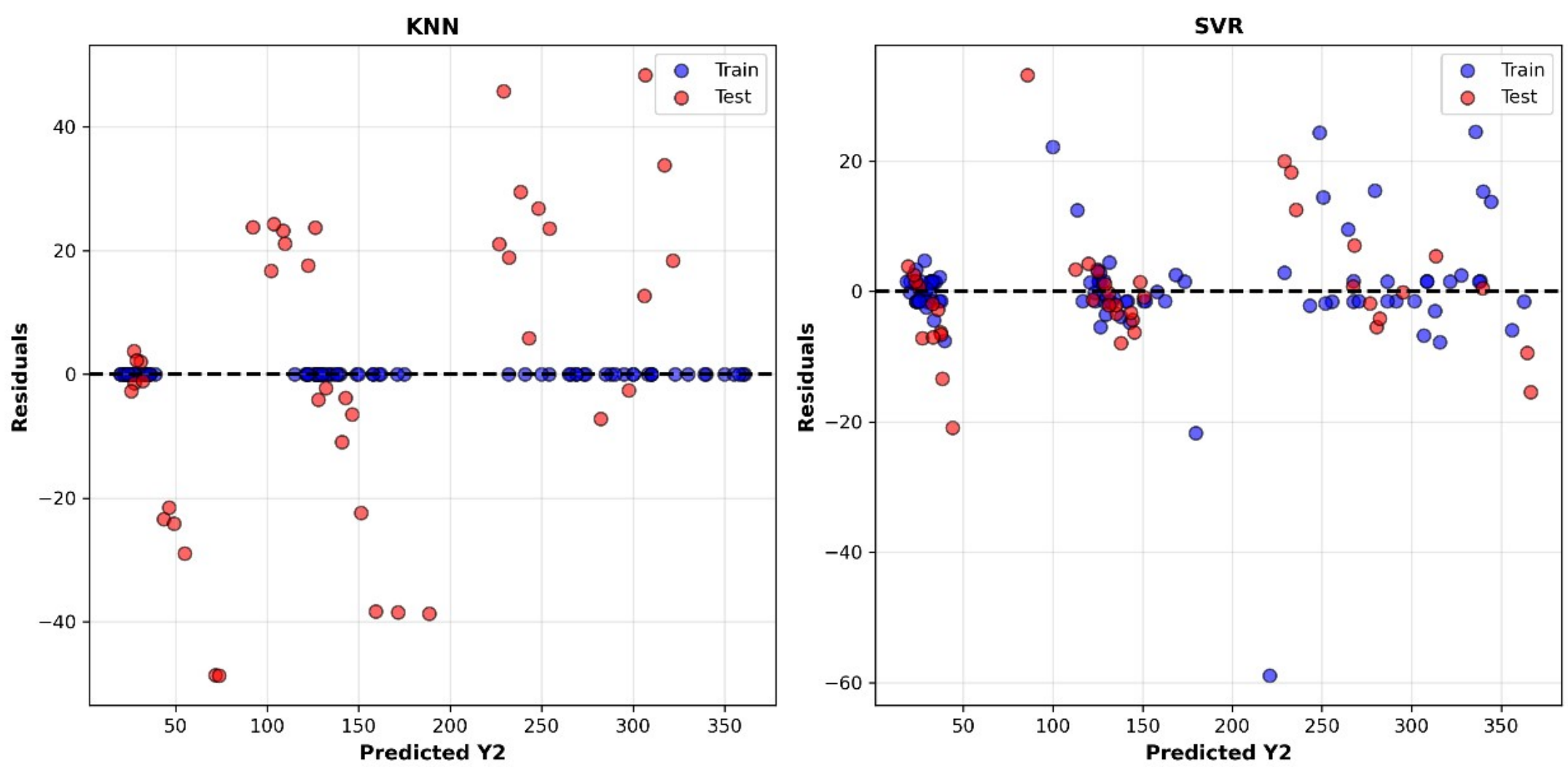

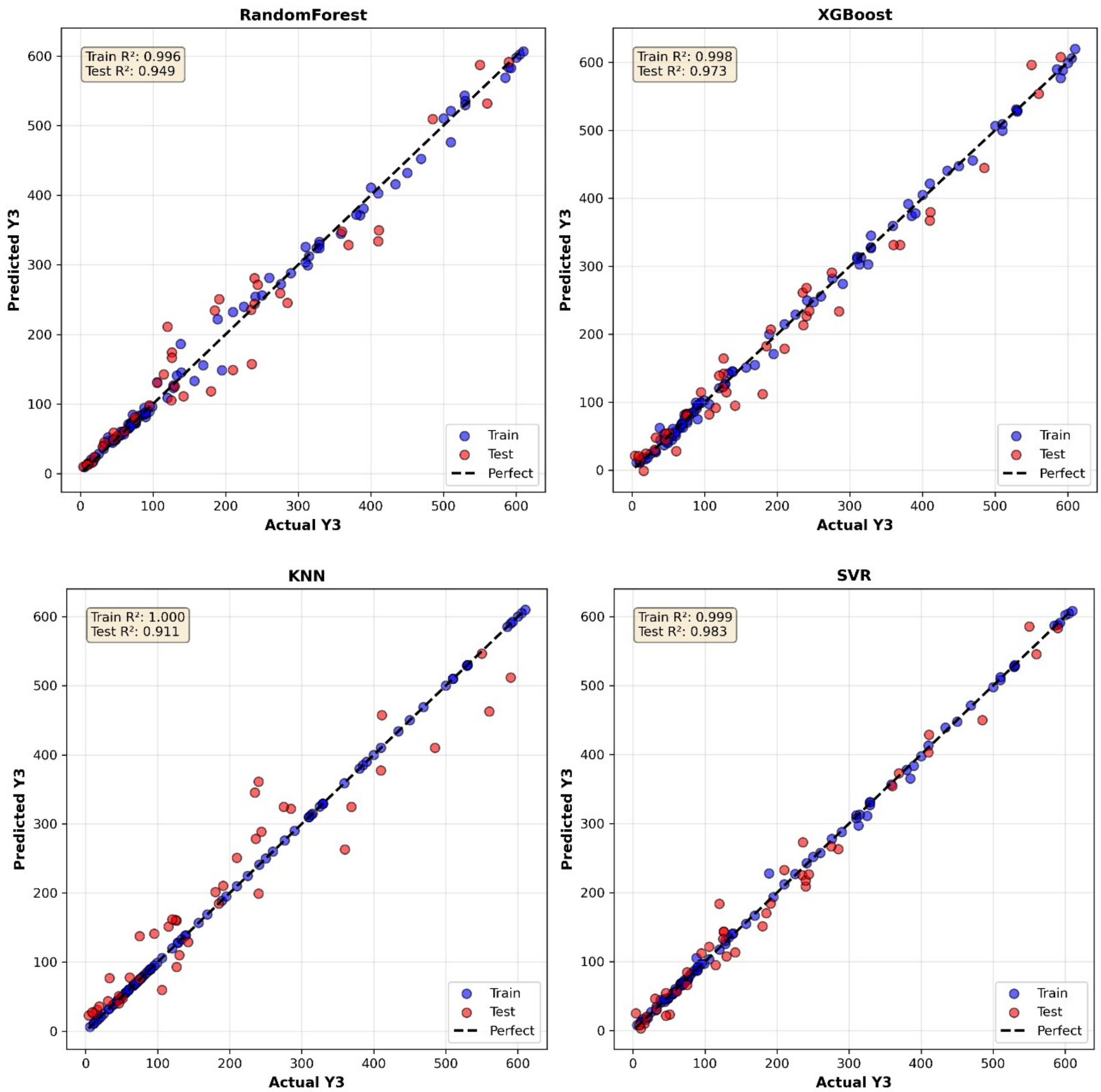

Figure S9. Predicted versus experimental values for Y3 obtained using Random Forest, XGBoost, KNN, and SVR models.

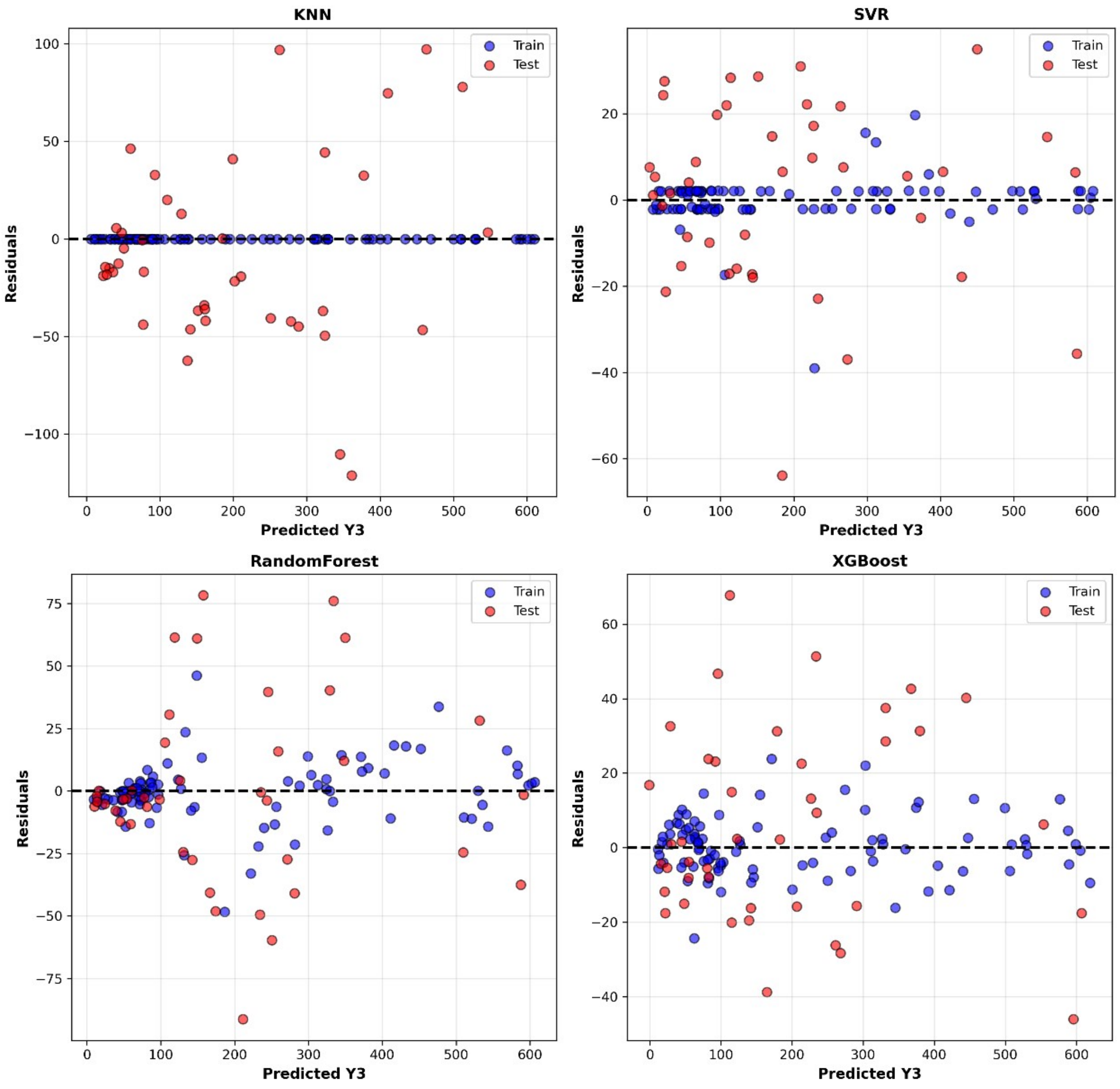
KNN
SVR
RandomForest
XGBoost
Train
Test
Residuals
Predicted Y3

Figure S10. Residual distributions for Y3 predictions using Random Forest, XGBoost, KNN, and SVR models.
